# Molecular insights into the fine-tuning of pH-dependent ArsR-mediated regulation of the SabA adhesin in *Helicobacter pylori*

**DOI:** 10.1093/nar/gkae188

**Published:** 2024-03-18

**Authors:** Anna Åberg, Pär Gideonsson, Abhayprasad Bhat, Prachetash Ghosh, Anna Arnqvist

**Affiliations:** Department of Medical Biochemistry and Biophysics, Umeå University, SE-90187 Umeå, Sweden; Department of Medical Biochemistry and Biophysics, Umeå University, SE-90187 Umeå, Sweden; Department of Medical Biochemistry and Biophysics, Umeå University, SE-90187 Umeå, Sweden; Department of Medical Biochemistry and Biophysics, Umeå University, SE-90187 Umeå, Sweden; Department of Medical Biochemistry and Biophysics, Umeå University, SE-90187 Umeå, Sweden

## Abstract

Adaptation to variations in pH is crucial for the ability of *Helicobacter pylori* to persist in the human stomach. The acid responsive two-component system ArsRS, constitutes the global regulon that responds to acidic conditions, but molecular details of how transcription is affected by the ArsR response regulator remains poorly understood. Using a combination of DNA-binding studies, *in vitro* transcription assays, and *H. pylori* mutants, we demonstrate that phosphorylated ArsR (ArsR-P) forms an active protein complex that binds DNA with high specificity in order to affect transcription. Our data showed that DNA topology is key for DNA binding. We found that AT-rich DNA sequences direct ArsR-P to specific sites and that DNA-bending proteins are important for the effect of ArsR-P on transcription regulation. The repression of *sabA* transcription is mediated by ArsR-P with the support of Hup and is affected by simple sequence repeats located upstream of the *sabA* promoter. Here stochastic events clearly contribute to the fine-tuning of pH-dependent gene regulation. Our results reveal important molecular aspects for how ArsR-P acts to repress transcription in response to acidic conditions. Such transcriptional control likely mediates shifts in bacterial positioning in the gastric mucus layer.

## Graphical Abstract

**Graphical Abstract:**
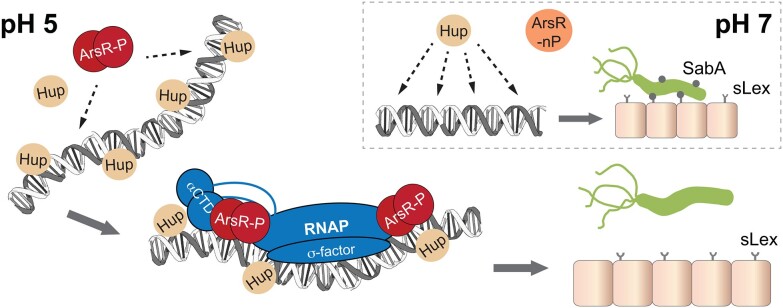

## Introduction

Pathogenic bacteria have evolved complex mechanisms to adjust their gene expression in response to its host environment. *Helicobacter pylori* persistently colonizes the human stomach, which increases the risk for developing peptic ulceration and gastric cancer in a significant part of the world's population (1,2). *H. pylori* are growing best at neutral pH but can readily adapt to the acidic conditions that they are encountering in the stomach. Most *H. pylori* bacteria are found deep in the mucus layer that protects the gastric epithelium, but a minor proportion are attached to the gastric epithelial cells (3,4). Thus, *H. pylori* must strictly control its gene expression in the pH gradient from the acidic conditions close to the lumen to the neutral pH close to the epithelial cells (5,6). The ability to adapt to the varying pH is critical for *H. pylori* to successfully colonize its host over the host's lifetime. A series of genes encode proteins with properties that are necessary for acid acclimation, like the urease enzyme that converts urea to ammonia and carbon dioxide that plays a central role in acid resistance (7–9). Other properties such as motility, adhesion, and chemotaxis are also affected by pH (5,10–12).

Bacterial two-component systems (TCSs) are the dominant form of signal transduction for adapting to environmental stimuli. The typical TCS consists of a membrane histidine kinase, which receives the specific environmental signal, and a cognate response regulator. The response regulator becomes phosphorylated by the sensor kinase, dimerizes, and then modulates target gene expression by binding to DNA (13–15). *H. pylori* has a small genome that only encodes three complete TCSs, two orphan regulators and one chemotactic system (16–18). The *H. pylori* acid-responsive regulon is under the control of the ArsRS TCS (17), including the sensory kinase ArsS (HP0165) and the OmpR-like response regulator ArsR (HP0166) (19,20). About 15% of the genes in *H. pylori* are differentially regulated at acidic pH and more than 100 genes are affected by the ArsRS TCS at acidic conditions, including increased expression of acid acclimation genes and repression of >30 cell envelope protein associated genes (19,21–27). Despite many years of research that have accumulated a wealth of knowledge, much of the regulatory details of the ArsRS regulon remain poorly understood (24,28).

To establish and maintain infection, adhesion to the target tissue is crucial, and close contact is a prerequisite for delivery of toxins and host-effector molecules (3). Bacteria often use multiple adhesion proteins that act in concert to accomplish adhesion in different environments. Both adhesion and motility in *H. pylori* are affected by pH (10–12), although they are oppositely regulated (24,28). The best-described *H. pylori* attachment proteins are the BabA adhesin, which mediates binding to the ABO/Lewis b (Leb) receptors in healthy gastric mucosa, and the sialic acid-binding adhesin SabA, which binds to sLex/a receptors in the inflamed gastric epithelium (29–32). BabA-mediated binding to the ABO/Leb receptors is sensitive to acidic pH, but not through changes in its expression. Instead, different pH values in the stomach select for BabA variants with different pH-dependent ABO/Leb binding affinities (33). During inflammation, expression of the sLex receptor increases on the gastric epithelium, and adherence of *H. pylori* is mediated via sLex-SabA binding (32,34). The progression to gastric cancer starts with the loss of acid-secreting parietal cells in the gastric corpus region, i.e. the middle part of the stomach, which results in an increase of pH (35,36), and the reprogramming and reorganization of epithelial cells in the gastric unit (37). In a mouse model, it was found that *H. pylori* colonized deep in the gastric glands, mediated by sLex-SabA binding and when the pH of the mouse stomach is increased using omeprazole, *H. pylori* colonization via sLex-SabA is extended to the corpus region (38). Expression of the SabA adhesin clearly decreases under acidic conditions (10,11,39), and expression is regulated by the ArsRS TCS (22,40,41). Thus, the molecular details on how pH-dependent *sabA* regulation occurs and the role for how ArsR operates remain unexplored.

Many host-adapted bacterial pathogens with small genomes - such as *H. pylori*, *Neisseria* spp. and *Haemophilus influenzae -*often contain numerous simple sequence repeats (SSRs) (42–44). The SSRs are mutational hot-spots, and they contribute to population heterogeneity and adaptation via so-called slipped-strand mispairing events (45–47). The location of SSRs in relation to a gene determines the outcome of the mispairing event. SSRs located in the promotor region or further upstream affect mRNA levels (48–50), SSRs in the 5′UTR affect translation initiation rates through binding of factors acting in *trans* such as sRNA (51), while SSRs located in the open reading frame lead to the phase-variable turning on/off of protein expression (32,40,52). We have previously shown that *sabA* mRNA levels are fine-tuned via a promoter-proximal SSR (48). The length of this SSR, a mononucleotide T-tract, varies between strains as well as among clones isolated from the same host. Changes in the local DNA structure and the axial alignment of UP-like elements relative to the core promoter thus modulate the transcriptional output.

In this work, we examine the mechanistic role of the global acid response regulator ArsR in the transcriptional regulation of one of the important outer membrane proteins of *H. pylori*, namely the SabA adhesin. We used recombinant ArsR in electrophoretic mobility shift assays (EMSAs), DNase I footprinting, and *in vitro* transcription (IVT) assays, and we showed that binding of ArsR is affected by intrinsic bends in the DNA and is restricted to two positions in the *sabA* promoter. Phosphorylated ArsR (ArsR-P) binds specifically to both positions, but repression of *sabA* transcription operates primarily through binding of ArsR-P to binding site II. With the help of the DNA-bending protein Hup, this affects the interaction of the RNA polymerase α-subunit with UP-like elements of the *sabA* promoter region, resulting in a decreased transcriptional output. Moreover, our results show that the length of the promoter-proximal SSR in the *sabA* promoter affects ArsR binding, transcriptional output, and pH-dependent regulation in *H. pylori*. This suggests that within-host genetic drift in combination with classical transcription factor activity guides the pH-dependent expression of the SabA adhesin in *H. pylori*, and possibly other outer membrane proteins. Thus, fine-tuning SabA expression to match available receptor structures and to fit local pH values is of great importance for allowing *H. pylori* to maintain a lifelong infection.

## Materials and methods

### Strains and growth conditions

The strains used in this study are described in Supplementary Table S1. *H. pylori* strains were grown on Brucella agar plates (BD Biosciences) supplemented with 10% citrated bovine blood (Svenska Labfab), 1% IsoVitox (Becton Dickinson), and an antibiotic mix (4 mg/l amphotericin B, 5 mg/l trimethoprim, and 10 mg/l vancomycin), or in culture medium containing Brucella broth (BD Biosciences), 1% IsoVitox, 10% fetal calf serum (Gibco), and an antibiotic mix (4 mg/l amphotericin B and 5 mg/l trimethoprim). Plates or broth were, when required, supplemented with streptomycin (100 mg/l), chloramphenicol (20 mg/l), and/or kanamycin (25 mg/l). Bacteria were grown at 37°C under microaerophilic conditions (5% O_2_, 10% CO_2_, and 85% N_2_). *Escherichia coli* strains were cultured in Luria broth (LB) agar at 37°C supplemented with carbenicillin (100 mg/l), chloramphenicol (20 mg/l), and/or kanamycin (25 mg/l). Growth was measured by OD_600_ using an Ultrospec2100 PRO spectrophotometer (Cytiva). For the acid stress experiments, *H. pylori* strains were grown in Brucella broth (pH 7.0) for 48 h to an OD_600_ of 0.2–0.3. To initiate the stress experiment, the cultures were split in two and 4 M sterile HCl was added to reach a pH of 5.0. For control experiments, an equal volume of sterile water was added to maintain a pH of 7.0. The cultures were incubated at 37°C for 24 h before the samples were collected. The pH of the cultures was measured at 0 min and after 24 h, and no change in the pH value was detected.

### Genetic techniques

Basic molecular genetic manipulations were performed essentially as described previously (53). Genomic *H. pylori* DNA was isolated from bacteria grown on a plate, as earlier described (54). Polymerase chain reactions (PCRs) were carried out according to the manufacturer's instructions using GoTaq polymerase (Promega) or Phusion Hot-Start DNA polymerase (Thermo Fisher Scientific) on an MJ PTC-200 thermal cycler (MJ Research). For isolation of plasmid DNA, the E.Z.N.A. Mini plasmid kit (Omega *bio-tek*) or NucleoBond Xtra Midi kit (Macherey-Nagel) was used. Purification of PCR products and DNA fragments was performed with QIAquick Gel or PCR clean-up kits (Qiagen) according to the manufacturer's instructions. Ligation and restrictions were made with enzymes from Thermo Fisher Scientific, according to the manufacturer's instructions. Plasmids and PCR products were sequenced by Eurofins Genomics.

### Plasmid construction

The plasmids and primers used are shown in Supplementary Tables S1 and S2, and all constructs were verified by sequencing. The *sabA* transcriptional *lacZ* fusion plasmid was constructed by cloning a PCR-amplified fragment (sabA-1/sabA-3) spanning 310 bp of the *sabA* promoter region and 8 bp of the CDS (–245 to +75) sub-cloned between the *EcoR*I/*BamH*I sites in pCR TOPO. Genomic DNA from SMI109 was used as the template. After sequencing, the DNA fragments were isolated by digestion with *Xho*I/*BamH*I and cloned between the *Xho*I/*Bgl*II sites in pBW to create the transcriptional fusion plasmid pAAG129.

The promoter regions of *ureA* and *sabA* were cloned into the *EcoR*I/*BamH*I sites of the IVT plasmid pTE103 and used as the template for EMSA, DNase I footprinting, and IVT. The *ureA* promoter was PCR-amplified from SMI109 (-250 to + 87) using ureA-1/ureA-2 primers to generate the plasmid pAAG261. The *sabA* promoter was PCR amplified from SMI109 (–245 to +75), G27 (–248 to +75) and 17875/sLex (–246 to +77) using sabA-1/sabA-3 primers to generate the plasmids pAAG264, pAAG265 and pAAG266, respectively. pAAG286 was constructed by cloning a PCR product generated by sabA-1/sabA-3 primers using chromosomal DNA from the SMI109 T_18_ variant. To generate the *sabA* promoters with scrambled AT DNA sequences in region 2, 3 or 4, stitch PCR was performed using P181/P182, P183/P184 or P173/P174 primers, respectively, and outer sabA-1/sabA-3 primers to generate the plasmids pAAG315, pAAG316, and pAAG267. pAAG334 containing the *sabA* promoter with scrambled regions 3 and 4 was constructed with P183/P184 and outer sabA-1/sabA-3 primers using the pAAG267 plasmid as the template. The plasmid containing part of the *sabA* ORF (+205 to +425) was constructed by cloning a PCR-generated product using the sabA-qP1F/sabA-qP1R primers and SMI109 chromosomal DNA into the *Sma*I site on pTE103 to generate plasmid pAAG328.

The expression plasmid for purification of His_6_-ArsR protein was constructed by PCR amplification of the *arsR* gene from SMI109 using the arsR-1/arsR-2 primers, which was then cloned into the *Nde*I/*BamH*I sites of the pET28a plasmid to generate plasmid pAAG156. The expression plasmid for purification of Hup-His_6_ was constructed by cloning PCR-amplified DNA from SMI109 using the hup-6/hup-7 primers, which was then cloned into the *Nde*I/*Xho*I sites of pET21a to generate the plasmid pAAG190.

### Construction of *H. pylori* strains

The plasmids and primers used are shown in Supplementary Tables S1 and S2, and all clones were verified by PCR and sequencing. SMI109P*sabA*::*lacZ* was constructed by transformation of strain SMI109 with the plasmid pAAG129. The plasmid gets incorporated into the chromosome of SMI109, leaving the *sabA* gene intact, and was verified by diagnostic PCR and immunoblot. SMI109Δ*arsS* was created by transformation of SMI109 with a Δ*arsS*::*rpsLcat* PCR fragment (P38/P43) generated by amplifying regions flanking the deletion by primers P38 with P40 and P41 with P43, amplifying the *rpsLCAT* cassette with P54 and P55, and joining these pieces with primers P38 and P43. The Δ*arsS*::*rpsLcat* PCR fragment was cloned into pUC19 to generate plasmid pAAG179. Insertion and deletion of the *arsS* gene in SMI109 was verified by PCR (Primers P59/P43). The isogenic clones with scrambled region 3* and 4* of P*sabA* were constructed by counterselection in strain SMI109, as previously described (48,55). The PCR fragment harboring the scrambled DNA was generated by stitch PCR using primers P68/P69 from two mutagenesis fragments made with primers P183/P184 (region 3*) and/or P173/P174 (region 4*), and introduced by transformation into SMI109 Δ*sabA*::*rpsLCAT*. Clones that regained SabA expression were identified by immunoblot with α-SabA antibodies (AK278), and the sequence was confirmed by PCR (primers P93/P96) and sequencing analysis.

### SDS-PAGE and immunoblot analysis

SDS-PAGE analysis was performed using NuPAGE Novex Bis-Tris protein gels (4–12%) using 1× MOPS SDS as the running buffer (Life Technologies). The gels were either stained with PAGE Blue protein stain (Thermo Fisher Scientific) or transferred to PVDF membranes using a Trans-Blot SD Semi-Dry Transfer Cell (Bio Rad). Immunoblot analysis was performed as previously described (52). Antibodies against SabA (AK278) and AlpB (AK262) (56) were used in combination with secondary goat anti-rabbit IgG-HRP (#31460, Invitrogen). Blots were developed with SuperSignal West Pico Chemiluminescent Substrate (Thermo Fisher Scientific) and detected on High Performance Chemiluminescence film (Cytiva) or on a ChemiDoc MP Imaging system (Bio Rad). Band densities were measured by ImageJ software (NIH) and normalized to the corresponding PAGE Blue-stained SDS-PAGE gels before relative protein expression levels were calculated.

### cDNA synthesis and reverse transcription quantitative PCR (RT-qPCR) analysis

Bacterial samples were collected in RNAprotect Bacteria reagent (Qiagen) according to the manufacturer's instructions, and total RNA was extracted using the RNeasy Mini Kit (Qiagen). Bacteria were lysed for 10 min in 1x TE (30 mM Tris–HCl [pH 8.0], 1 mM EDTA) containing 0.75 mg/ml lysozyme (Thermo Fisher Scientific) and 1 mg/ml Proteinase K (Qiagen). Contaminating DNA was removed using the TURBO DNA-free kit (Invitrogen). The quality and concentration of the total RNA was measured on a micro-spectrophotometer (Nanodrop ND-1000, Thermo Fisher Scientific). cDNA synthesis was performed using a Transcriptor First Strand cDNA Synthesis kit (Roche Applied Science) as described previously (48).

RT-qPCR was used to determine the mRNA levels of *sabA, ureA* and *hup* in different *H. pylori* strains, and the primers used are listed in Supplementary Table S2. As reference genes, the expression levels of *alpB* and *ppk* were analyzed simultaneously. For the experiment shown in Supplementary Figure S4B, *rrnA* and *gyrA* were used as reference genes. The cDNA synthesis and RT-qPCR analysis were performed in accordance with the MIQUE guidelines (57). RT-qPCR reactions were set up, run, and analyzed as described previously (48) using 2× FastStart Essential Green Master (Roche Applied Science) and a LightCycler 96 instrument (Roche Applied Science). The mRNA concentrations of each gene were calculated using efficiency-corrected Cq values and were normalized to the geometric mean concentration of the reference genes. The normalized ratios were later used to compare mRNA levels of target genes between different strains and/or conditions and to calculate relative differences. The primer efficiencies were determined using standard curve analysis and were as follows: *sabA-1* 1.98, *sabA-2* 1.99, *ureA* 1.97, *hup* 1.95, *ppk* 1.99, *alpB* 1.93, *gyrA* 1.96 and *rrnA* 1.90. *sabA-2* primers were used when *sabA* expression was analyzed in different *H. pylori* strains (Figure 4C).

### Primer extension

Total RNA was extracted using the SDS/hot phenol method as previously described (48). The primer used for primer extension analysis was the [γ^32^P]ATP kinase-labeled sabA-8 primer (Supplementary Table S2). The labeled primer was annealed to 20 μg of total RNA, and primer extension reactions were performed using 1 U of AMV reverse transcriptase (Promega) as previously described (58). The reaction products were separated by electrophoresis on a 6% polyacrylamide/8.3 M urea sequencing gel. The bands were visualized after exposure to a PhosphoImager cassette and scanned with a Typhoon scanner 9400 (Cytiva). A + G Maxam Gilbert sequencing reactions using the same primer as for the primer extension were loaded as the size marker (59).

### β-Galactosidase assay

β-Galactosidase activity measurements were performed as described by Miller (60) and calculated as Miller Units (MU) using the formula $\frac{{1000\ \cdot \ (O{D}_{420}\ - \ ( {1.75\ \cdot \ O{D}_{550}} )}}{{t\ \cdot \ v\ \cdot \ O{D}_{600}}}$, where *t* is the reaction time (in minutes) and *v* is the volume (in milliliters) of bacterial culture used for each reaction. Data are shown as the mean values with standard deviations of at least three independent experiments.

### Protein expression and purification

All protein overexpressions were made in BL21(DE3)/pLysS cells (Novagen). Fresh transformants were inoculated in 500 ml LB media and grown to an OD_600_ of 0.5 before induction with IPTG. Induction was performed with 1 mM IPTG for 3h at 37°C (His_6_-ArsR) or with 0.05 mM IPTG (Hup-His_6_) for 16 h at 22°C. All protein purifications steps were performed at 4°C with pre-chilled solutions. Protocols were adapted from manufacturer's recommendations for the resin and/or columns used for each purification as described below. The purity of the recombinant proteins in the collected fractions was analyzed by SDS-PAGE analysis and PAGE Blue protein staining (Thermo Fisher Scientific). Protein concentrations were determined using Qubit Protein Assay Kits and a Qubit 4 fluorometer (Thermo Fisher Scientific), and all proteins were kept at –80°C until analysis. The His-tags remained on the proteins during the analysis they was used for.

N-terminally His_6_-tagged ArsR_WT_ was overexpressed from pAAG156. The cell pellet was resuspended in 7 ml per gram of bacterial pellet of FP buffer (50 mM Tris–HCl [pH 7.5], 150 mM KCl, 10 mM MgCl_2_, 10% glycerol) containing EDTA-free protease inhibitor cocktail (Roche Applied Science), disrupted by sonication, and centrifuged at 27 000 × *g* for 25 min. The cleared supernatant was applied to a column with 1 ml cobalt-chelated agarose (HisPur Cobalt resin, #89964, Pierce) equilibrated with 10 ml FP buffer. The resin was washed with 10 ml FP buffer containing 10 mM imidazole, and the proteins were eluted with 3 ml FP buffer containing 20 mM imidazole and with 3 ml containing 100 mM imidazole. The eluted protein (3 ml) was dialyzed twice against 2 l storage buffer (50 mM Tris [pH 7.5], 50 mM KCl, 10 mM MgCl_2_, 20% glycerol and 1 mM DTT) for at least 4 h each. His_6_-ArsR protein (20 μM) was phosphorylated *in vitro* by incubating the protein in phosphorylation buffer (50 mM Tris–HCl [pH 7.5], 20 mM MgCl_2_, 50 mM KCl) in the presence of 50 mM lithium potassium acetyl phosphate (#A0262, Sigma Aldrich) for 30 min at 37°C prior to being used in EMSA, DNase I footprinting, or IVT.

C-terminally His_6_-tagged Hup was expressed from pAAG190, and cell pellets were resuspended in 7 ml per gram of bacterial pellet of Buffer A (50 mM sodium phosphate [pH 7.4], 25 mM NaCl) containing 5 mM imidazole and EDTA-free protease inhibitor cocktail (Roche Applied Science), disrupted by sonication, and centrifuged at 20 000 × *g* for 25 min. The supernatant was applied to a column with 1 ml nickel-chelated NTA agarose (HisPur Ni-NTA resin, #88221, Pierce) equilibrated with 10 ml Buffer A. The resin was washed with 10 ml Buffer A + 20 mM imidazole, and proteins were stepwise eluted with 3 ml batches of Buffer A containing 50, 150 and 300 mM imidazole. The eluted protein (3 ml) was dialyzed twice against 2 l storage buffer (20 mM sodium phosphate [pH 7.4], 50 mM KCl, 20% glycerol and 1 mM DTT) for at least 4 h each.

### Electrophoretic mobility shift assay (EMSA)

Linear DNA fragments were generated by high-fidelity PCR using IVT plasmids as templates (Supplementary Table S1) and the 486/485 primer pair (Supplementary Table S2) where the 485 primer was labeled with tetrachlorofluorescein (TET). The plasmids used as templates for each PCR are written in the figure legends of each figure, as well as the exact concentration of DNA and proteins used. The binding reactions were performed in a final volume of 15 μl using a final concentration of 5–7 nM TET-labelled DNA and 0–4 μM of protein mixed in buffer F (50 mM Tris-Ac [pH 7.5], 100 mM potassium glutamate, 20 mM magnesium acetate and 5% glycerol) containing 0.1 μg Poly dI-dC (#81349, Sigma Aldrich), and incubated for 30 min at 25°C. The samples were separated on 8% 1× TBE polyacrylamide gels. The gels were scanned on a glass plate, and bands were visualized in the Amersham Typhoon 5 (Cytiva) using a 532 nm laser and a 570 nm emission filter and 100 micron resolution. The amount of non-shifted DNA was quantified in each lane using the Analysis toolbox of the ImageQuantTL 10.2 software (Cytiva) with separate background corrections for each sample. The amount of shifted DNA was calculated from the average measurements of the control samples (containing only DNA). The *K*_d(app)_ (the amount of protein needed to shift 50% of the DNA) was calculated from the amount of shifted DNA for each experiment when a series of protein concentrations was used. The average and standard deviation of at least three independent experiments is presented in Figures 2A, 4A and 7E.

For the EMSA shown in Figure 2B, the competitor DNA was generated by high-fidelity PCR using IVT plasmids as the templates (Supplementary Table S1) and the 486/485 primer pair (Supplementary Table S2). Plasmids pAAG328 and pAAG261 were used for the *sabA* ORF DNA and for P*ureA* DNA, respectively. The binding reactions were performed as described above with 5 nM TET-labelled P*sabA* DNA, 0 or 2 μM non-phosphorylated or phosphorylated His_6_-ArsR, and 0–20 nM competitor DNA. For the EMSA shown in Figure 5C, methyl green (#M8884, Sigma Aldrich) or netropsin (#N9653, Sigma Aldrich) was added to the binding reactions at a final concentration of 0–100 μM. The binding reactions were performed as described above with 5 nM TET-labelled P*sabA* DNA and 0 or 2 μM non-phosphorylated or phosphorylated His_6_-ArsR.

### DNase I footprinting

Linear DNA fragments were generated by high-fidelity PCR using IVT plasmids as templates (Supplementary Table S1) and the 486/485 primer pair (Supplementary Table S2). One of the primers used was TET-labelled to detect binding to each DNA strand, with 485-TET being used for the coding strand and 486-TET being used for the non-coding strand. Recombinant proteins were purified, and His_6_-ArsR was phosphorylated as described above. Binary complexes were formed by incubating with a final concentration of 25 nM TET-labeled DNA and 10 μM His_6_-ArsR or 300 nM σ^70^-RNAP (#M0551S, New England Biolabs). Binding reactions were performed in a final volume of 20 μl in buffer F (50 mM Tris-Ac [pH 7.5], 100 mM potassium glutamate, 20 mM magnesium acetate, and 5% glycerol) containing 0.1 μg Poly dI-dC for 30 min at 25°C. The reactions were digested by adding 3 μl of 0.2 U/μl DNase I (#AM2235, Ambion) diluted in 10 mM Tris–HCl [pH 7.5], 25 mM MgCl_2_ and 10 mM CaCl_2_ followed by incubation for 60–90 s at 25°C. The reactions were terminated by the addition of 200 μl dissociation solution (0.5 M ammonium acetate, 10 mM magnesium acetate, 1 mM EDTA, 0.1% SDS, 50 μg/ml salmon sperm DNA (#15632-001, Invitrogen) and 0.3 μg/ml glycogen (#R0561, Thermo Fisher Scientific), and the samples were extracted with 200 μl phenol before the DNA was ethanol precipitated at –80°C. DNA pellets were recovered by centrifugation at 17 000 x *g* for 30 min at 25°C, washed with 70% ethanol, dried, and resuspended in 10 μl formamide loading buffer (95% [v/v] formamide, 10 mM EDTA, 0.1% bromophenol blue). The samples were denatured at 95°C for 5 min and analyzed on a denaturing gel consisting of 8% polyacrylamide, 7 M urea, and 25% formamide. A + G Maxam Gilbert sequencing reactions (59) of the same DNA fragments were loaded alongside the samples. The gels were scanned between glass plates, and bands were visualized on an Amersham Typhoon 5 (Cytiva) using a 532 nm laser and a 570 nm emission filter and 100 micron resolution. Quantitative analysis of the DNase footprint was performed using the Gel and Blot tool in the ImageQuantTL 10.2 software (Cytiva). Digital images of the gels were quantitatively analyzed, and the background was subtracted using the rolling ball settings. Data were plotted as densities of DNase footprint bands (intensity) versus the migration of bands (position) counted from the top to the bottom of the gel.

For the DNase I footprint gel in Supplementary Figure S1, binding reactions with 25 nM DNA and 10–20 μM non-phosphorylated or phosphorylated His_6_-ArsR were performed in a final volume of 20 μl in buffer F containing 0.1 μg Poly dI-dC for 30 min at 25°C. The reactions were digested by adding 3 μl of 0.2 U/μl DNase I followed by incubation for 60–90 seconds at 25°C. The reactions were terminated by the addition of 5 μl MassRuler DNA loading dye (#R0621, Thermo Fisher Scientific) and directly loaded onto 8% TBE PAGE gels. The gels were stained with a 1/12000 dilution of GelRed (#41003, Biotium), and the DNA was visualized by UV light. Non-shifted and shifted DNA were excised from the gel, crushed with a 1.5 ml pestle, and extracted by the addition of 200 μl dissociation solution for 2h at 60°C. Gel pieces were removed by centrifugation at ≥14 000 x *g* for 5 min, and samples were run through an MC centrifugal filter (#UFC30GV25, Millipore). The samples were extracted with 200 μl phenol before the DNA was ethanol precipitated at –80°C. DNA pellets were recovered by centrifugation at 17 000 x *g* for 30 min at 25°C, washed with 70% ethanol, dried, and resuspended in 10 μl formamide loading buffer. The samples were denatured at 95°C for 5 min and analyzed on a denaturing gel of 8% polyacrylamide, 7 M urea and 25% formamide gel. A + G Maxam Gilbert sequencing reactions (59) of the same DNA fragments were loaded alongside the samples. The protocol was adapted from Hsieh *et al.* (61).

### *In vitro* transcription assay (IVT)

IVT assays were performed using supercoiled plasmid DNA based on the pTE103 plasmid (Supplementary Table S1). *E. coli* σ^70^-RNAP holoenzyme was obtained from New England Biolabs (#M0551S), and recombinant proteins were purified and phosphorylated as described above. Multiple-round assays (15 μl reactions) were performed at 37°C in a transcription buffer (50 mM Tris-acetate [pH 7.5], 50 mM potassium glutamate, 20 mM magnesium acetate and 5% glycerol) containing 0.1 mg/ml bovine serum albumin (#AM2616, Ambion) with 0–10 nM σ^70^-RNAP and 0.5–2 nM plasmid DNA. The plasmids used as templates and exact concentration of DNA, RNAP and regulatory proteins are specified in the figure legends of corresponding figure. The DNA template was incubated with regulatory protein (or appropriate storage buffer) for 8 min prior to the addition of σ^70^-RNAP in order to allow time for open-complex formation. Transcription was initiated by adding a mixture of NTPs (#R0481, Thermo Fisher Scientific, including ATP (final concentration 0.5 mM), GTP and CTP (final concentration 0.2 mM each), UTP (final concentration 0.8 mM)) and [α-^32^P]-UTP (5 μCi at > 3000 Ci mmol^−1^; Perkin Elmer). Reactions were terminated after 15 min by addition of 15 μl stop/load buffer (80% [v/v] formamide, 100 mM Tris–HCl [pH 8.0], 99 mM boric acid, 2 mM EDTA, 0.5% xylene cyanole, and 0.25% bromophenol blue). Transcripts were analyzed on 6% polyacrylamide gels containing 7 M urea, and the bands were visualized after exposure to a PhosphoImager cassette and scanned with a Typhoon 9400 (Cytiva) or Amersham Typhoon 5 (Cytiva). Band intensities were quantified using the Gel and Blot tool in the ImageQuantTL 10.2 software (Cytiva) with rolling ball background corrections.

For the time-course experiment (Figure 8D), 0.5 nM plasmid DNA was mixed with regulatory proteins (storage buffer, 125 nM Hup-His, 1 μM phosphorylated His_6_-ArsR, or both) at 37°C for 8 min prior to the addition of 10 nM σ^70^-RNAP to allow for open-complex formation. Transcription was initiated by adding a mixture of NTPs, and 15 μl samples were removed and mixed with 15 μl stop/load buffer after 1, 2.5, 5 and 10 min. Transcripts were analyzed on 6% polyacrylamide gels containing 7 M urea, and the bands were visualized and quantified as described above.

For the single and multiple-round experiment (Figure 8E), 0.5 nM plasmid DNA was mixed with regulatory proteins (storage buffer, 125 nM Hup-His_6_, 1 μM phosphorylated His_6_-ArsR, or both) at 37°C for 10 min prior to addition of 10 nM σ^70^-RNAP to allow for open-complex formation. Single-round transcription was initiated by adding a mixture of NTPs containing 125 μg/ml Heparin (#H4784, Sigma Aldrich) and after 10 min 15 μl stop/load buffer was added. Multiple-round transcription was initiated by adding a mixture of NTPs, and reactions were terminated after 10 min by the addition of 15 μl stop/load buffer. Transcripts were analyzed on 6% polyacrylamide gels containing 7 M urea, and the bands were visualized and quantified as describe d above.

### Bioinformatics and statistical analysis

We used the DNASTAR Lasergene software MegAlign for all DNA analyses, and we used GraphPad Prism 10 for the statistical analyses. We used unpaired two-tailed non-parametric Mann–Whitney U-test for single-pair comparisons. Differences were considered significant when the *P-*value was below 0.05. Significance levels are marked with * <0.05, ** <0.01, *** <0.001, and **** <0.0001, and non-significant is marked with ns if *P*> 0.05.

## Results

### pH-dependent repression of *sabA* expression occurs at the transcriptional level

**Figure 1.**
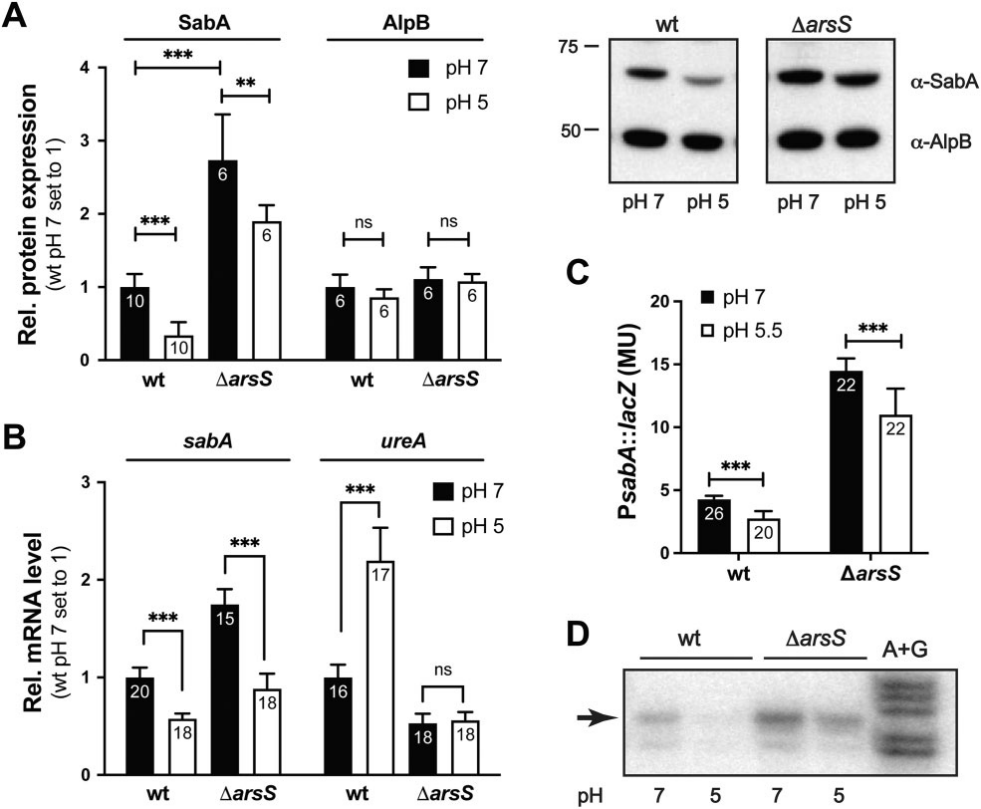
SabA expression is regulated by acidic pH at the transcriptional level. (**A**) Change in expression of SabA and AlpB protein in SMI109 wt and in a strain lacking ArsS sensor kinase (Δ*arsS*) after acid stress. Cultures were grown in Brucella broth to OD_600_ of 0.2–0.3 before the pH shift. Samples were collected 24 h after the shift to pH 5 (white bars) or as a control after growth at pH 7 (black bars). SabA and AlpB protein expression was analyzed by immunoblotting, quantified, and plotted relative to the expression observed in SMI109 wt at pH 7 (for each protein). The image to the right shows one representative immunoblot from the four independent experiments performed. The number of data points each average is based on are shown in the bars of the diagram. The statistical analysis was performed with the Mann-Whitney U-test (*P*< 0.001, ***; *P*< 0.01, **; *P*> 0.05, ns). (**B**) The effect of pH on *sabA* and *ureA* mRNA levels in SMI109 wt and Δ*arsS* strains as measured by RT-qPCR. Cultures were grown as described in (**A**). Data from three biological replicates, each with two technical replicates, were used for the quantification. The number of data points each average is based on are shown on the bars of the diagram. Statistical analysis was performed with the Mann–Whitney U-test (*P*< 0.001, ***; *P*< 0.01, **). (**C**) The effect on P*sabA* activity after acid stress as measured by β-galactosidase activity in the SMI109 P*sabA*::*lacZ* strain. Cultures of the wt and Δ*arsS* strains were initially grown as described in (A). The pH was kept at pH 7 as a control (black bars) or shifted to pH 5.5 (white bars) for 24 h. Quantifications were made from four independent experiments, and the number of data points each average is based on are written on the bars of the diagram. The statistical analysis was performed with the Mann–Whitney U-test (*P*< 0.001, ***). (**D**) Primer extension analysis of *sabA* using RNA isolated from SMI109 wt and Δ*arsS* after acid stress. Cultures were grown as described in (A). Total RNA was mixed with radiolabeled sabA-8 primer, and the samples were separated in a denaturing polyacrylamide gel. Maxam and Gilbert DNA sequencing reactions (A + G) were performed using the same primer and were loaded as size controls.

Because the expression levels of more than 100 genes in *H. pylori* are affected by pH via the ArsRS TCS and because the accumulated knowledge on the role of ArsRS remains limited (24,62), we aimed to gain deeper mechanistic insights into ArsR-mediated pH-dependent transcriptional regulation. More than 30 genes are repressed by the ArsRS system and/or pH, and a majority of those encode surface-exposed proteins (10,11,22,24). For this study, we selected the surface exposed SabA adhesin as our model system and the Swedish *H. pylori* gastric cancer isolate SMI109 was used as the main reference strain. To calibrate experimental setup and our model strain with previously published results, we cultured SMI109 (wt) strain and a deletion mutant of the *arsS* (Δ*arsS*) gene at pH 7, which corresponds to the pH close to the epithelial cells. Subsequently, we induced a shift to pH 5, corresponding to the pH of the mucus layer closer to the lumen or the corpus stomach region. Initially, we monitored the effect on pH on expression levels of the SabA adhesin and the outer membrane protein AlpB, using immunoblot analyses. The expression of the SabA adhesin decreased after the shift to pH 5, whereas the expression of AlpB remained unaffected (Figure 1A). Additionally, RT-qPCR confirmed that it is the *sabA* mRNA level that was repressed at pH 5 (Figure 1B). In the Δ*arsS* strain, both SabA protein and *sabA* mRNA levels were clearly derepressed, indicating that ArsS inhibits *sabA* expression (Figure 1A,B). This derepression occurred both at neutral and acidic pH. Moreover, in the Δ*arsS* strain, the pH-dependent repression of *sabA* remained, as previously observed in other studies (22,24). As a control, the expression of *ureA* was simultaneously analyzed by RT-qPCR, and here *alpB* was used as one of the reference genes. As anticipated, the *ureA* mRNA levels were clearly increased in a pH-dependent and ArsS-mediated manner in our experimental setup (Figure 1B), like previously described. Furthermore, results from test with a *sabA*::*lacZ* promotor fusion construct introduced into the SMI109 wt and Δ*arsS* strains, were fully consistent with the above described findings (Figure 1C). Using primer extension analysis, we found that transcription from the *sabA* promoter was affected by pH and that the same transcriptional start site was used in the Δ*arsS* strain as in the wt (Figure 1D). As previously published (63), no additional transcriptional start site was observed upstream of the T-tract in the *sabA* promoter region. On the basis of the above described results, we conclude that our experimental setup and *H. pylori* strain align with most of the previously published findings. Furthermore, we also conclude that both the pH-dependent repression, as well as the ArsS-mediated repression, of *sabA* expression occur at the transcriptional level.

### Phosphorylation of ArsR increases DNA binding at two sites in the *sabA* promoter

**Figure 2.**
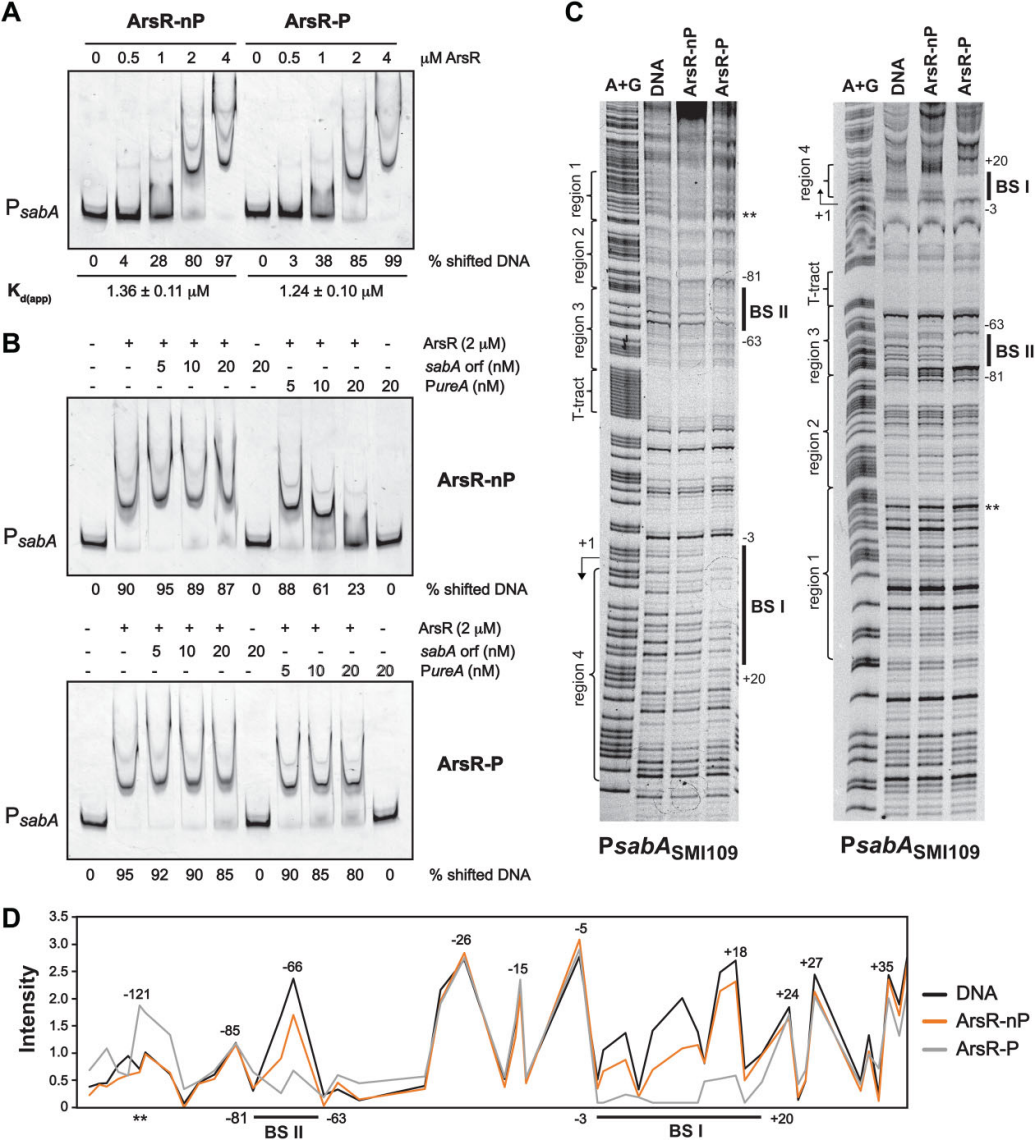
ArsR binds to P*sabA* DNA at two different positions. (**A**) Binding of His_6_-ArsR to P*sabA* DNA analyzed by EMSA. A total of 7 nM TET-labelled P*sabA* DNA (407 bp PCR; primers 486/485-TET; template pAAG264) was mixed with increasing concentrations (0–4 μM) of non-phosphorylated His_6_-ArsR (ArsR-nP) or phosphorylated His_6_-ArsR (ArsR-P). The amount of non-shifted DNA was quantified, and the percentage of shifted DNA was calculated. Average calculations of % shifted DNA from three independent experiments are shown below the picture with a standard deviation of ±3%. The *K*_d(app)_ (amount of protein needed to shift 50% of the DNA) was calculated separately for ArsR-nP and ArsR-P for each experiment. The mean and standard deviation are shown below the picture. Statistical analysis of the *K*_d(app)_ between ArsR-nP and ArsR-P, with the Mann–Whitney U-test showed *P*> 0.05 (ns). (**B**) Binding of His_6_-ArsR to P*sabA* DNA in the presence of non-specific or specific competitor DNA. A total of 5 nM TET-labelled P*sabA* DNA (407 bp, same as in A) was mixed with 2 μM ArsR-nP or ArsR-P and 0–20 nM non-specific (*sabA* ORF; 301 bp PCR; primers 486/485; template pAAG328) or specific competitor DNA (P*ureA*; 424 bp PCR; primers 486/485, template pAAG261) was added to the reactions. The amount of non-shifted DNA was quantified, and the percentage of shifted DNA was calculated. The average calculations of the percentage of shifted DNA from two independent experiments are shown below each picture with standard deviations of ±2%. (**C**) DNase I footprint analysis of His_6_-ArsR binding to P*sabA* DNA from SMI109. The gel image to the left is with DNA of the coding strand (407 bp PCR; primers 486/485-TET; template pAAG264), and the gel image to the right is with DNA of the non-coding strand (407 bp PCR; primers 486-TET/485; template pAAG264). Transcriptional start site (+1) and T-tract are marked to the left of the gel images, as well as the regions described in Figure 3A. The Maxam and Gilbert DNA sequencing reaction (lane A + G) shows the sequence of the DNA used. A total of 25 nM DNA was mixed with protein storage buffer (lane DNA), 10 μM ArsR-nP (lane ArsR-nP), or 10 μM ArsR-P (lane ArsR-P). Binding sites are marked by solid lines, and the nucleotide positions of the binding sites are shown to the right of the gel images. The hypersensitive site is marked with **. Here is shown one representative gel of at least two independent experiments. (**D**) Line representation of the protection pattern of His_6_-ArsR on P*sabA*_109_ shown in C (black, free DNA; orange, ArsR-nP; grey, ArsR-P). The densities of the DNase I footprint bands (indicated by intensity) were quantified and plotted as migration (from the top to bottom of the DNase footprint). Nucleotide positions are written on top of the peaks, and the two ArsR binding sites are marked by solid lines below the diagram.

Deletion of the *arsR* gene is lethal (17), so studies of ArsR as a transcriptional regulator need to be approached biochemically. The pH-dependent gene regulation has been suggested to occur via the phosphorylation and dephosphorylation of ArsR (17), and that the phosphorylated ArsR would bind to DNA and regulates gene expression. However, other studies have also suggested that there could also be a phosphorylation-independent regulatory mechanism via the ArsRS TCS (62). We purified recombinant His_6_-tagged ArsR_WT_ (ArsR) and phosphorylated the protein *in vitro* and measured its binding to P*sabA* using EMSA. The EMSA experiments with TET-labelled P*sabA* DNA showed a clear DNA shift with both non-phosphorylated (ArsR-nP) and phosphorylated protein (ArsR-P) (Figure 2A). We found no significant difference in the protein concentration needed to shift 50% of the DNA (*K*_d(app)_) for ArsR-nP versus ArsR-P. This shows that the DNA binding could occur independently of phosphorylation. To validate the binding, a non-specific competitor consisting of part of the *sabA* ORF was added to the EMSA reactions (Figure 2B). This DNA did not affect the binding of either ArsR-nP (Figure 2B, top) or ArsR-P (Figure 2B, bottom), even when a four-fold higher concentration was used. Using a specific competitor consisting of P*ureA* DNA, a clear difference was observed. This DNA could out compete the binding of ArsR-nP but not the binding of ArsR-P in the concentration range used (Figure 2B). Suggesting that ArsR-P binds to P*sabA* DNA with higher sequence specificity than ArsR-nP.

To precisely map the ArsR binding sites in the P*sabA* region, we employed a high-resolution DNase I footprint assay using TET-labelled DNA and the ArsR-P protein. A clear protected region was observed (−3 to +20, BS I), which partially overlapped with a previously suggested ArsR binding site (+3 to +39) (64). Interestingly, a second protected site located upstream of the promoter-proximal T-tract (−81 to −63, BS II) was observed in P*sabA* (Figure 2C, left). When the opposite DNA strand was analyzed, DNase I footprints were observed at the same positions (Figure 2C, right). In EMSA we also observed binding of ArsR-nP, so we decided to also test the effect of phosphorylation in DNase footprint assays. However, using the ArsR-nP protein we did not see any visible protected area on either of the DNA templates used (Figure 2C). Furthermore, the band intensities of the DNase footprint shown in Figure 2C (left) was quantified to compare the binding of ArsR-nP and ArsR-P (Figure 2D). A clear difference between DNA alone (black line) and ArsR-P (grey line) was observed at the two binding sites (Figure 2D). We now observed that ArsR-nP (orange line) bound preferably to BS I (Figure 2D), although the binding was weaker than for ArsR-P (grey line). For the rest of the nucleotide positions, the band intensities were almost identical. A hypersensitive site was detected at position −121 in the sample with ArsR-P suggesting that the DNA conformation was altered upon binding of ArsR-P (Figure 2C, D, **). To increase the number of DNA-protein complexes of ArsR-nP prior to the footprint analysis, DNA-protein complexes digested with DNase I were separated by PAGE and then extracted, precipitated, and loaded onto the sequencing gel (61). Two different concentrations of protein, both non-phosphorylated and phosphorylated, were used together with a DNA control (Supplementary Figure S1). Although the signal was weaker than earlier after the long processing of the samples, the results now showed even more clearly that ArsR-nP protein bound weakly to BS I but not at all to BS II, whereas ArsR-P bound to both sites (Supplementary Figure S1). A line representation of the protection pattern was also made, and we found that a higher protein concentration yielded stronger binding (Supplementary Figure S1). These results showed that ArsR-P has two binding sites in P*sabA* – BS I (−3 to +20), overlapping with the transcriptional start site, and BS II (−81 to −63), upstream of the T-tract. The binding was specific for ArsR-P to BS II, and phosphorylation of ArsR increased binding to BS I.

**Figure 3.**
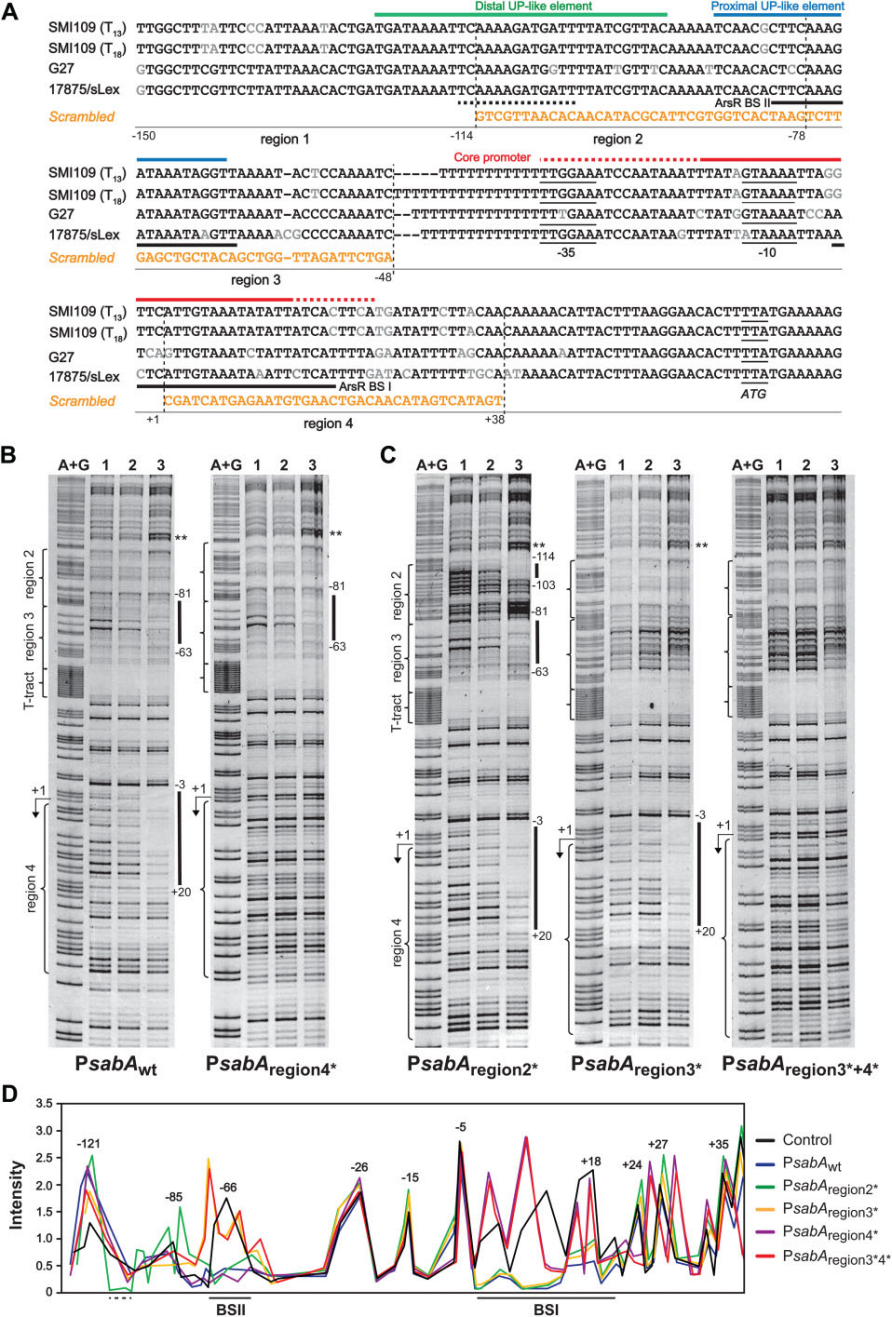
Sequences with long AT stretches are required for specific ArsR binding. (**A**) Sequence alignment of the *sabA* promoter region of the *H. pylori* strains used in this study; SMI109 (wt, T_13_), SMI109 (T_18_), G27 and 17875/sLex. The −35 and −10 regions are marked, as well as the transcriptional start site (+1). The solid black lines mark the two ArsR binding sites found by DNase I footprinting, namely BS II (−81 to -63) and BS I (−3 to + 20). The AT stretches in regions 2–4 were scrambled to reduce the length of AT in the DNase I footprint analysis (shown in B and C). The sequences of the scrambled regions are shown in orange text below each region. The additional binding site (−117 to −104) observed with region 2* DNA (shown in C) is marked by a dotted line. The distal and proximal UP-like elements and the core promoter where RNAP binds (Supplementary Figure S5A) are marked with green (distal), blue (proximal), and red (core) lines. The nucleotide positions relative to the +1 transcriptional start site are shown below indicating the sizes of the different regions. (**B**) DNase I footprint analysis of His_6_-ArsR binding to P*sabA* (407 bp PCR; primers 486/485-TET) wt DNA (template pAAG264) and scrambled region 4* (template pAAG267). Transcriptional start site (+1) and T-tract are marked along the left side, as well as the regions described in (A). Maxam and Gilbert DNA sequencing reaction (lanes A + G) showing the sequence of DNA used. DNA was mixed with protein storage buffer (lane 1), 10 μM ArsR-nP (lane 2), or 10 μM ArsR-P (lane 3). Binding sites are marked by solid lines along the right side. The image shows one representative gel of at least three independent experiments. (**C**) DNase I footprint analysis of His_6_-ArsR binding to scrambled region 2*, 3*, and 3*+4*. The same setup was used as described in (B), but different templates were used to make P*sabA* region 2* (template pAAG315), region 3* (template pAAG316), and region 3*+4* (template pAAG334). (**D**) Line representation of the protection pattern of ArsR-P on P*sabA* with scrambled region 2*, 3*, 4* and 3*+4* shown in (B) and (C). The control represents the DNase I footprint analysis of P*sabA*_wt_ DNA without any other protein. The densities of the DNase I footprint bands were quantified and plotted as a migration (from the top to bottom of the DNase footprint). Nucleotide positions are written on top of the peaks, and the two ArsR binding sites are marked by solid lines below the diagram. The full line representation of each lane in the DNase I footprint gels shown in (B) and (C) can be found in Supplementary Figure S2B.

When analyzing the DNA sequences, we found a high frequency of longer AT stretches in ArsR binding sites on P*sabA* DNA and these were also highly conserved between different *H. pylori* strains (Supplementary Figure S2A and Supplementary Table S3). No clear consensus binding sites has yet been described for ArsR (19,20,65,66) and looking further into the proposed ArsR binding sites in 11 different promoter regions, we found that most binding sites have several long AT stretches (Table 1). To establish if the binding of ArsR is dependent on these AT stretches, the P*sabA* DNA sequence was scrambled to reduce the lengths of the AT stretches (Figure 3A). Part of the P*sabA* DNA (−150 to +38) used for the binding studies was divided into four regions with equal size before the DNA sequence in region 2–4 was scrambled. The region −47 to +1, containing the T-tract and core promoter, was excluded (Figure 3A). These scrambled DNA fragments were used in DNase I footprint assays together with ArsR-P. When the region containing BS I was scrambled (region 4*, +2 to +38), no binding to BS I was observed, while binding to BS II remained (Figure 3B, right; and Figure 3D purple line). When the region containing BS II was scrambled (region 3*, −77 to −48), no binding to BS II was observed, while binding to BS I remained (Figure 3C, middle; and Figure 3D yellow line). Finally, when both regions were scrambled, no binding of ArsR-P was observed to P*sabA* DNA (Figure 3C, right; and Figure 3D red line). When we scrambled the region upstream of BS II (region 2*, −113 to −78), binding to BS I and BS II was not affected; however, an additional binding site appeared at position −113 to −103 (Figure 3C, left; and Figure 3D green line). Subsequently, binding of ArsR-nP was also analyzed to the different DNA templates, but no differences from previous observations were detected (Figure 3B–D and Supplementary Figure S2B). This suggests that ArsR-P binding to one binding site is not required for binding to the other site, and vice versa. Further, long AT stretches of DNA are important for specific binding.

**Table 1.** ArsR binding sites in different promoter regions in *H. pylori*

Positively regulated genes						
Gene	Name	Binding site (AT^GC^)	Length / Max AT		Position	Reference
*hp0119*	*hp0119*	3^2^3^2^7^3^7	27 bp	7 bp	−50 to −20	(65)
*hp0294*	*amiE*	6^1^5^1^6^1^4^1^5^1^9^3^4^1^1^1^7	56 bp	9 bp	−70 to −15	(22)
*hp1238*	*amiF*	6^1^1^3^5^1^1^1^7^1^4^1^3^1^2	38 bp	7 bp	−50 to −20	(22)
*hp0073*	*ureA -1*	2^1^4^4^7^3^1^1^2^1^6^2^	34 bp	7 bp	−140 to −110	(20)
	*ureA -2*	2^1^17^1^5^1^2^3^2^1^1^1^5^1^3^1^5^1^1	54 bp	17 bp	−70 to −20	(20)
*hp0071*	*ureI*	6^1^12^1^1^1^6^1^7^1^5^1^4^1^	48 bp	12 bp	−50 to −1	(20)
*hp0869*	*hypA -1*	3^1^6^1^1^1^13	24 bp	13 bp	−165 to −141	(23)
	*hypA -2*	3^1^8^1^11^1^5^1^6^1^7^2^1^1^1	50 bp	11 bp	−111 to −62	(23)
*hp1186*	β-CA	2^1^3^2^8^1^1^1^10^1^4^2^2^1^6^2^3	50 bp	10 bp	−144 to −95	(23)
*hp1408*	*hp1408*	3^1^2^1^5^2^3^1^3^1^3	27 bp	5 bp	−50 to −20	(65)
**Negatively regulated genes**						
**Gene**	**Name**	**Binding site** **(AT^GC^)**	**Length / Max AT**		**Position**	**Reference**
*hp0166*	*arsR*	3^2^3^1^5^2^4	20 bp	5 bp	+10 to + 30	(65)
*hp0725*	*sabA-2*	^1^2^1^3^1^7^2^2	19 bp	7 bp	−81 to −63	*This study*
	*sabA-1*	2^1^3^1^12^1^	23 bp	12 bp	−3 to +20	*This study*
*hp1399*	*rocF*	^1^5^1^2^1^10^2^4^2^12^2^2^1^8^2^4^1^2	62 bp	12 bp	−65 to −5	(22)

Analysis of AT and GC content in ArsR binding sites reveals a preference for binding to long AT stretches. Published ArsR binding sites in 11 different promoter regions were compared and analyzed for their AT and GC content (see text for references). The binding site length variations are shown in the right column, and the maximum length of the AT stretches is shown in the left column.

### ArsR binding is modified by changes in the T-tract length, which in turn alters the pH-dependent repression of *sabA* expression

**Figure 4.**
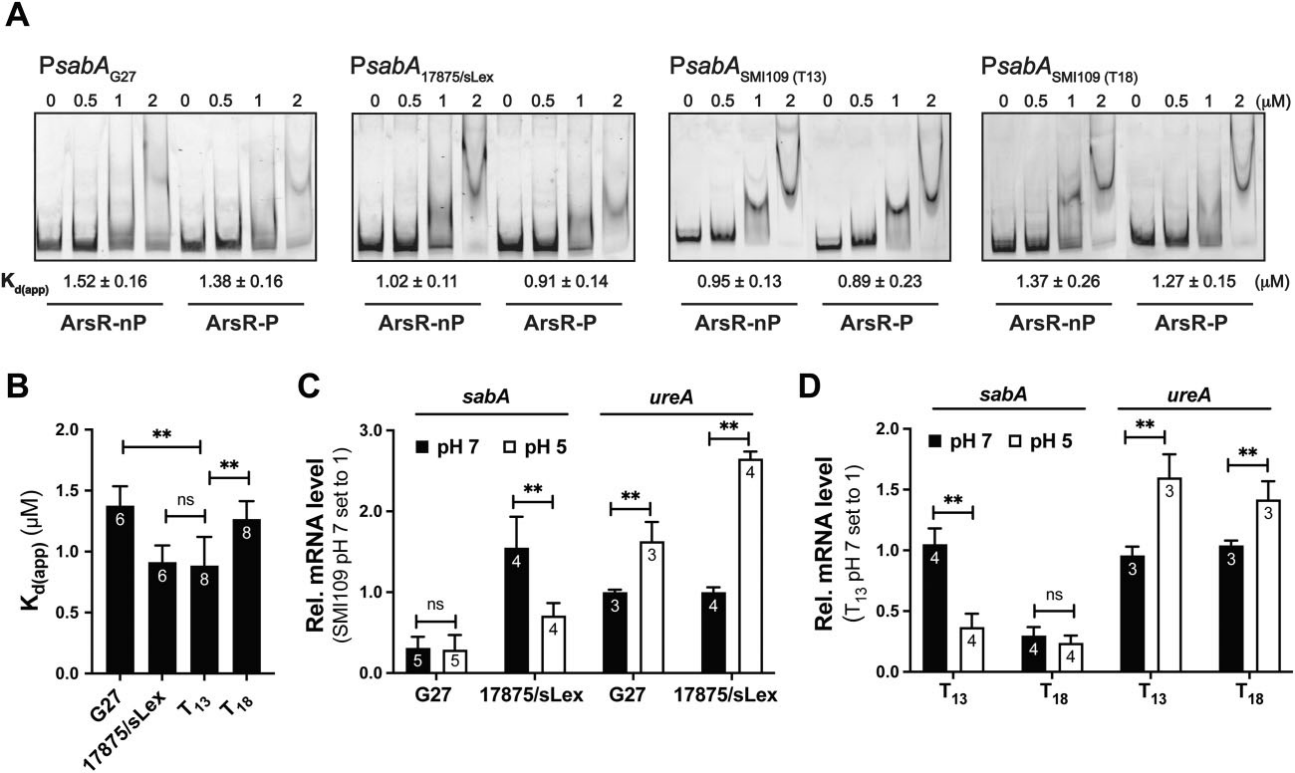
Small sequence variations and T-tract length affect ArsR binding to P*sabA* and the pH-dependent regulation of *sabA* expression. (**A, B**) Binding of His_6_-ArsR to P*sabA* DNA from different strains or with different T-tract length analyzed by EMSA. A total of 5 nM TET-labelled P*sabA* DNA (407–412 bp, 486/485-TET primers) with G27 (template pAAG265), 17875/sLex (template pAAG266), SMI109 T_13_ (template pAAG264), or SMI109 T_18_ (template pAAG286) was mixed with 0–2 μM ArsR-nP or ArsR-P. (A) The images show one representative gel from three to four independent experiments. The amount of non-shifted DNA was quantified, and the percentage of shifted DNA was calculated. Calculations of *K*_d(app)_ for each template DNA with ArsR-nP or ArsR-P were made for each gel image. The mean and standard deviation of four independent experiments is shown below each gel picture. (B) *K*_d(app)_ for ArsR-P to each P*sabA* DNA are plotted in the bar diagram and the number of data points each average is based on are shown on the bars of the diagram. Statistical analysis was performed with the Mann–Whitney U-test showing (*P*< 0.01,**, *P*> 0.05, ns). (**C, D**) Effect of pH on *sabA* and *ureA* mRNA levels in different *H. pylori* strains after acid stress and measured by RT-qPCR. Cultures of G27 or 17875/sLex (C) or isogenic variants of SMI109 with different T-tract lengths (D) were grown as described in Figure 1A. Data from two biological replicates, each including two technical replicates, were used for the quantification. The number of data points each average is based on are shown on the bars of the diagram. Statistical analysis was performed with the Mann–Whitney U-test (*P*< 0.01, **; *P*> 0.05, ns).

*H. pylori* has high intraspecies genetic variability, especially in genes encoding outer membrane proteins (67,68). Bioinformatic analysis of P*sabA* DNA in a series of published *H. pylori* strains revealed that the highest sequence variability is found in region 4 of the promoter, which contains ArsR BS I. The sequence variation between strains in region 3, containing ArsR BS II, is lower (Figure 3A and Supplementary Figure S2A). We selected two additional *H. pylori* strains (G27 and 17875/sLex) to see if the binding position and strength of ArsR to P*sabA* DNA is affected by these small variations in DNA sequence. To test if the binding position changes, DNase footprint assay was performed using TET-labelled DNA from strains G27 and 17875/sLex. No major difference in the position of the two ArsR binding sites was observed (Supplementary Figure S3A-B) compared to P*sabA* from SMI109 (Figure 2C). For G27, ArsR-P bound to −5 to +19 (BS I) and −79 to −58 (BS II) of P*sabA* DNA (Supplementary Figure S3A), and for 17875/sLex ArsR-P bound to −6 to +19 and −78 to −63 (Supplementary Figure S3B). An enhanced cleavage site was observed when ArsR-P was bound to 17875/sLex P*sabA* DNA at position +5, suggesting that binding of ArsR-P might be different to this DNA compared to the other two strains DNA (Supplementary Figure S3B). EMSA was used to analyze the binding strength of ArsR-nP and ArsR-P to P*sabA* DNA fragments from different strains. ArsR-nP and ArsR-P showed significantly stronger binding to P*sabA* DNA from 17875/sLex and SMI109 (T_13_) compared to P*sabA* from G27 (Figure 4A, B).

Another difference in P*sabA* DNA sequence from these different strains is the length of their promoter-proximal T-tracts where strain SMI109 has T_13_, G27 has T_17_ and 17875/sLex has T_15_ (Figure 3A). We have previously shown that the length of the T-tract affects the DNA curvature and influences SabA expression by changing the spatial alignment of the UP-like elements and the core promoter (48). Because we show here that the two ArsR binding sites are located on each side of this T-tract, we decided to analyze whether the T-tract length also could affect the binding of ArsR-P. In the DNase I footprint assay, we observed protected regions at the same positions in both P*sabA* SMI109 T_13_ (Figure 2C, D) and P*sabA* SMI109 T_18_ DNA (Supplementary Figure S3C), suggesting that ArsR-P binds to the same positions independently of T-tract length. Using EMSA, we tested the binding strength of ArsR to P*sabA* DNA with different T-tract lengths. Interestingly, both ArsR-nP and ArsR-P bound better to P*sabA* SMI109 T_13_ than to P*sabA* SMI109 T_18_ DNA (Figure 4A, B), similar to what we observed when comparing binding to G27 and 17875/sLex P*sabA* DNA. This suggests that variation in the T-tract length and thereby the DNA structure impacts the binding strength of ArsR-P.

As described above that the binding strength of ArsR-P to P*sabA* is altered when using DNA from different *H. pylori* strains. We decided to test if pH-dependent repression of *sabA* expression also varies, similar to the binding of ArsR-P *in vitro*. We analyzed *sabA* expression in the low-expressing strain G27 and the high-expressing strain 17875/sLex by exposing them to acidic stress followed by gene expression analyses using RT-qPCR. We detected the expected difference in *sabA* expression between these strains at pH 7, with 17875/sLex displaying high and G27 low *sabA* mRNA levels, also when bacteria were grown in broth (Figure 4C). Moreover, a clear repression of *sabA* expression at pH 5 was observed in the 17875/sLex strain (Figure 4C), similar to what we observed with SMI109 (a high-expressing strain) (Figure 1B). Interestingly, when the G27 strain was analyzed, no repression of *sabA* expression was observed at pH 5 (Figure 4C). To eliminate the impact of an unknown regulator as the cause for the difference in pH-dependent regulation, a pair of isogenic T-variants of strain SMI109 (T_13_ and T_18_) were exposed to acid stress conditions, and *sabA* mRNA levels were analyzed using RT-qPCR (Figure 4D). Corroborating our previous results (48), we could observe a clear difference in *sabA* mRNA levels in the T_18_ variant (low-expressing) compared to the T_13_ variant (high-expressing) (Figure 4D). Interestingly, we did not observe any pH-dependent regulation in the low-expressing T_18_ variant as we did for the high-expressing T_13_ variant (Figure 4D). As a control, we followed the expression of *ureA* in the same strains and saw a clear increase in all strains at pH 5 (Figure 4C, D). Thus, variations in the DNA sequence in different strains and in the T-tract length are likely to fine-tune ArsR-mediated pH-dependent regulation of SabA expression.

### The nucleoid-associated Hup protein impacts the pH-dependent regulation of *sabA* expression

**Figure 5.**
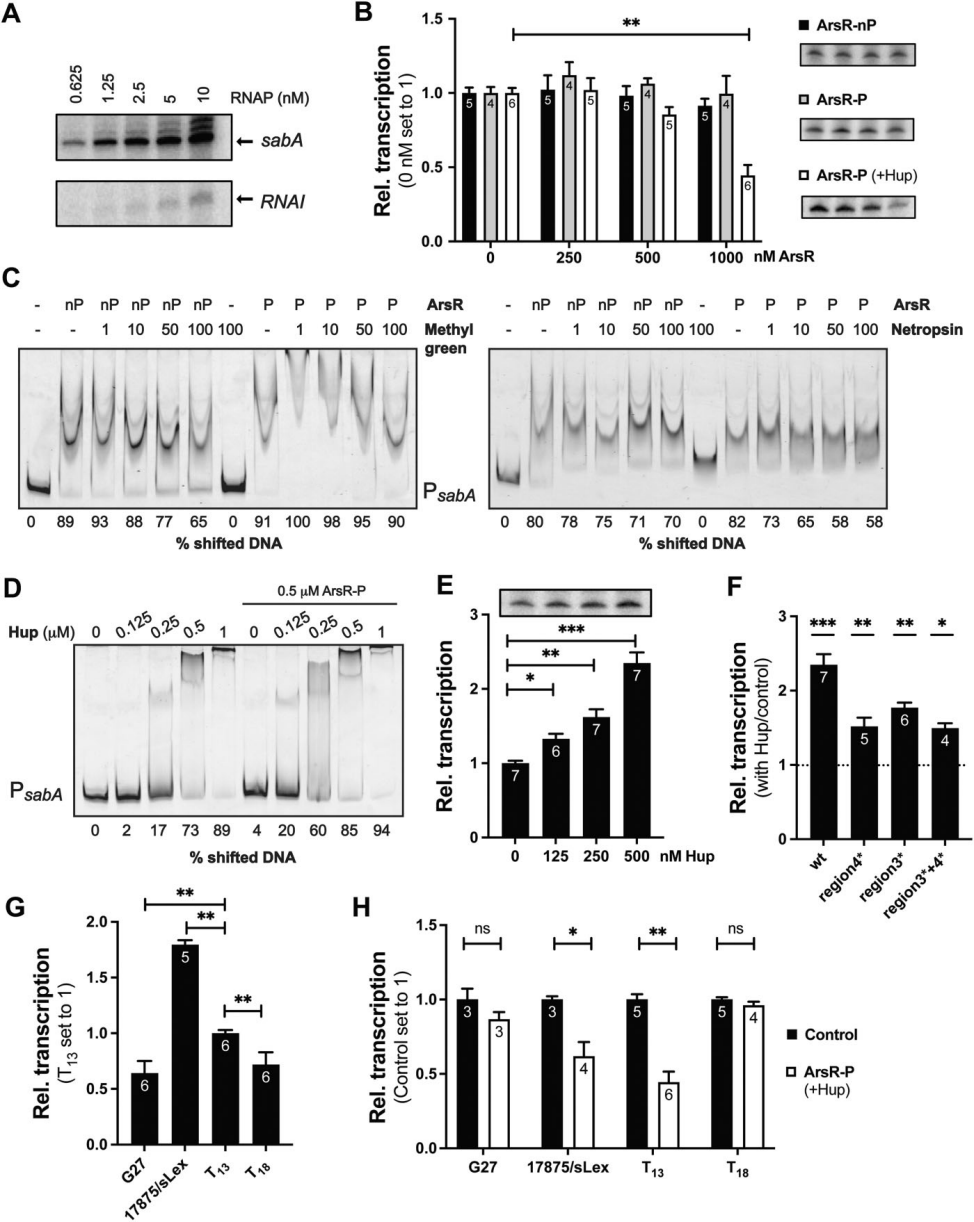
Transcription of *sabA* is enhanced by Hup and repressed by ArsR-*P in vitro*. (**A**) Set up of the *in vitro* transcription (IVT) assay with RNAP and P*sabA*_wt_ DNA. A total of 2 nM of supercoiled template (pAAG264) and 0–10 nM of *E. coli* σ^70^-RNAP (New England Biolabs) was used in multiple-round IVT assays run at 37°C. (**B**) IVT assay with P*sabA*_wt_ DNA and increasing concentrations of His_6_-ArsR. A total of 0.5 nM of supercoiled template (pAAG264) and 10 nM of RNAP was used in multiple-round IVT assays run at 37°C. A total of 0–1 μM ArsR-nP or 0–1 μM ArsR-P was added to each reaction. To one set of ArsR-P, 125 nM Hup-His_6_ was added to each reaction. The data were plotted relative to the transcript level with 0 nM ArsR set to 1. Black bars show the relative transcript level with ArsR-nP, grey bars show the relative transcript level with ArsR-P without Hup, and the white bars show the relative transcript level with ArsR-P and Hup. Images show one representative gel from at least four separate experiments, and the bar graph represents the combined results. The number of data points for each reaction are written in the bars of the diagram. Statistical analysis was performed on the raw data using the Mann–Whitney U-test (*P*< 0.01, **). (**C**) Binding of His_6_-ArsR to P*sabA* DNA in the presence of methyl green and netrospsin. A total of 5 nM TET-labelled P*sabA* DNA (407 bp, same as in Figure 2A) was mixed with 2 μM ArsR-nP (nP) or 2 μM ArsR-P (P). A total of 0–100 μM methyl green (left) or 0–100 μM netropsin (right) was added to each reaction. The amount of non-shifted DNA was quantified, and the percentage of shifted DNA was calculated. The mean values of the percentage of shifted DNA from three independent experiments are shown below each picture with a standard deviation of ±2%. (**D**) Binding of Hup-His_6_ to P*sabA* DNA as analyzed by EMSA. A total of 7 nM of TET-labelled P*sabA* DNA (407 bp, same as in Figure 2A) was mixed with increasing concentrations of Hup (0–1 μM) in the absence or presence of 0.5 μM ArsR-P. The amount of non-shifted DNA was quantified, and the percentage of shifted DNA was calculated. The mean values of the percentage of shifted DNA from four independent experiments are shown below each picture with a standard deviation of ±3%. (**E**) IVT assay with P*sabA*_wt_ DNA and increasing concentrations of Hup-His_6_. A total of 0.5 nM template DNA (pAAG264) was mixed with 10 nM RNAP and 0–500 nM Hup. The experiment was performed as described in B. The amount of transcript was plotted relative to 0 nM Hup set to 1. The image shows one representative gel, and the bar graph shows the combined results from four independent experiments. The number of data points for each reaction is shown on the bars of the diagram. Statistical analysis was performed on the raw data using the Mann–Whitney U-test (*P*< 0.001, ***; *P*< 0.01, **; *P*< 0.05, *). (**F**) IVT assay using P*sabA* templates with scrambled DNA sequences in region 2–4 and Hup-His_6_. A total of 0.5 nM template DNA (P*sabA*_wt_; pAAG264, P*sabA*_region3*_; pAAG316, P*sabA*_region4*_; pAAG267, P*sabA*_region3*+4*_; and pAAG334) was mixed with 10 nM RNAP and 0 or 500 nM Hup. Experimental procedures were as described in (B). The relative amount of transcript formed (500 nM/0 nM Hup) was calculated and plotted for each template DNA. The bar graph shows the combined results from at least two independent experiments. The number of data points for each reaction is shown on the bars of the diagram. Statistical analysis was performed on the raw data using the Mann–Whitney U-test (*P*< 0.001, ***; *P*< 0.01, **; *P*< 0.05, *). (**G**) IVT assay with P*sabA* templates from different strains (G27 and 17875/sLex) or with different T-tract lengths (SMI109/T_13_ and T_18_). A total of 0.5 nM template (P*sabA*_G27_; pAAG265, P*sabA*_17875/sLex_; pAAG266, or P*sabA*_SMI109_; pAAG264 and P*sabA*_T18_; pAAG286) and 10 nM of RNAP were used in multiple-round IVT assays run at 37°C. The amount of transcript was quantified and plotted relative to the amount obtained with SMI109/T_13_ that was set to 1. The bar graph shows the combined results from three independent experiments. The number of data points for each reaction is shown on the bars of the diagram. Statistical analysis was performed on the raw data using the Mann–Whitney U-test (*P*< 0.01, **). (**H**) IVT assay with His_6_-ArsR and P*sabA* templates from different strains (G27 and 17875/sLex) or with different T-tract lengths (SMI109/T_13_ and T_18_). A total of 0.5 nM template (P*sabA*_G27_; pAAG265, P*sabA*_17875/sLex_; pAAG266, or P*sabA*_SMI109_; pAAG264 and P*sabA*_T18_; pAAG286) was mixed with 10 nM of RNAP, 125 nM Hup, and 0 or 1 μM ArsR-P. Experimental procedures was as described in (B). The amount of transcript formed without ArsR-P (black bars, control) was set to 1 for each template DNA. The bar graph shows the combined results from at least two independent experiments. The number of data points for each reaction is shown on the bars of the diagram. Statistical analysis was performed on the raw data using the Mann–Whitney U-test (*P*< 0.01, **; *P*< 0.05, *; *P*> 0.05, ns).

To study the effect of ArsR on *sabA* transcription initiation, we established an IVT assay using *E. coli* σ^70^-RNA polymerase (RNAP) and P*sabA* DNA from the SMI109 strain. We have previously shown that *E. coli* σ^70^-RNAP recognizes and binds strongly to P*sabA* DNA (48). Using multiple-round assays that allow for re-initiation, transcription was observed already when using 0.625 nM of RNAP and 2 nM of supercoiled template (Figure 5A). Our aim with establishing the IVT assay was to study the mechanistic effect of ArsR-P on *sabA* transcription. However, the addition of increasing concentrations of ArsR-nP (Figure 5B, black bars) or ArsR-P (Figure 5B, grey bars) to the reactions resulted in negligible effects on P*sabA* transcription *in vitro*.

Gene expression controlled by OmpR-like regulators has been shown to be affected by DNA topology (69,70), and the long AT stretches, including the T-tract, important for ArsR binding might affect the local DNA structure by introducing intrinsic DNA bends (71). To test if binding of ArsR is affected by DNA topology, we used the DNA structuring drugs methyl green and netropsin in EMSA with P*sabA* DNA. Methyl green binds to the major groove of AT-rich DNA (72) and netropsin binds to the minor groove of AT-rich DNA, thus altering the DNA structure (73). The EMSA reactions were performed with ArsR-nP or ArsR-P at a concentration that shifted almost all P*sabA* DNA. The result showed that in the presence of methyl green, binding of ArsR-nP was affected but not the binding of ArsR-P (Figure 5C, left). It also appeared that low concentrations of the drug increased binding of ArsR-P to P*sabA* DNA. Netropsin, on the other hand, affected the binding of ArsR-P but had little effect on the binding of ArsR-nP (Figure 5C, right). Interestingly, these results suggest that ArsR-nP mainly interacts with the major groove of DNA whereas ArsR-P binds to the minor groove. Based on the findings that DNA-structuring drugs affect the binding of ArsR, we asked if a DNA structuring protein is required to observe an effect of ArsR-P on *sabA* transcription *in vitro*.

In *H. pylori*, only two nucleoid-associated proteins, Hup and HP0119, and a few proteins that affect supercoiling have been described (74–76). Previous studies have suggested that Hup plays a role in pH regulation by regulating the expression of urease in *H. pylori* (77,78) and the expression of genes involved in various types of stresses in *E. coli* (79,80). Because Hup binds to DNA in a non-specific manner, preferentially to AT-rich sequences and thereby introducing and/or stabilizing DNA bends (80–82), we started by using EMSA to determine whether recombinant Hup-His_6_ (Hup) could bind to P*sabA* DNA. Indeed, we found that Hup bound directly to P*sabA* DNA in a concentration-dependent manner, and binding was further increased in the presence of low concentrations of ArsR-P (Figure 5D). Nevertheless, when binding was tested with the DNase I footprint assay, no visible footprint was seen with Hup alone, and no change was seen in the ArsR-P binding sites when analyzed together (Supplementary Figure S4A). To test for a direct effect of Hup on *sabA* transcription, we analyzed transcriptional output with increasing concentrations of Hup in the IVT assay. We observed a clear increase in *sabA* transcription with increasing concentrations of Hup (Figure 5E). We also analyzed the effect of Hup on transcription from a *sabA* promoter containing scrambled DNA sequences in region 3* and region 4*, which resulted in decreased activation of transcription relative to P*sabA*_wt_ containing long AT stretches (Figure 5F).

To further evaluate how well the IVT assay reflects changes in the *sabA* mRNA levels observed in *H. pylori*, we analyzed transcription from P*sabA* DNA from different strains (G27 and 17875/sLex) or with different T-tract lengths (T_13_ and T_18_ variants of SMI109). Transcription from both *sabA* low-expressing promoters (G27 and T_18_) was significantly lower than transcription from the *sabA* high-expressing promoters (17875/sLex and T_13_) (Figure 5G), similar to the changes in mRNA levels seen in *H. pylori* (Figure 4C, D). Because Hup affects transcription from the *sabA* promoter *in vitro*, we decided to preincubate P*sabA* DNA with low concentrations of Hup together with increasing concentrations of ArsR-P to see if Hup could enhance the ArsR-dependent repression of *sabA* transcription. We now observed significant repression of *sabA* transcription *in vitro* (Figure 5B, white bars). Furthermore, we also analyzed if the repressing effect of ArsR-P on *sabA* transcription was affected like pH-dependent regulation was in *H. pylori*. Using IVT in the presence of Hup, we found that ArsR-P repressed transcription from the *sabA* high-expressing 17875/sLex and T_13_ promoters but not from the *sabA* low-expressing G27 and T_18_ promoters (Figure 5H). These results corroborated the results in *H. pylori* (Figure 4C, D). Clearly, the DNA structuring protein Hup is required to observe the repressing effect of ArsR-P on *sabA* transcription *in vitro*.

**Figure 6.**
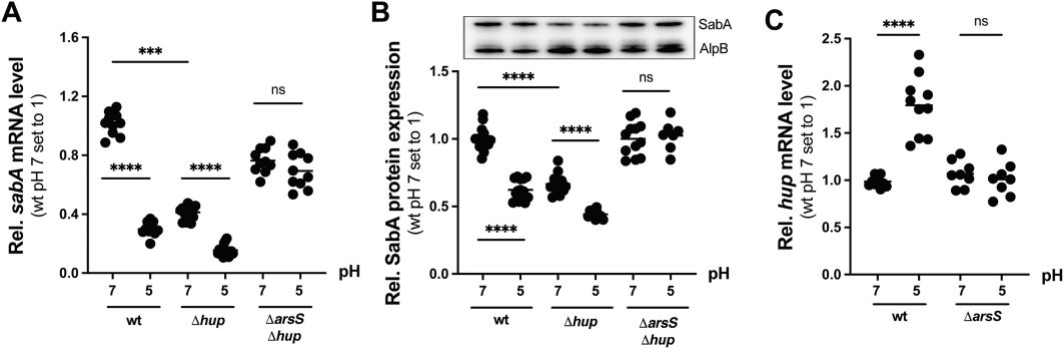
pH-dependent regulation of *sabA* expression is abolished in a Δ*arsS*Δ*hup* strain. (**A**) The effect of acid stress on *sabA* mRNA levels in the SMI109 wt, Δ*hup*, and Δ*arsS* Δ*hup* strains as measured by RT-qPCR. Cultures were grown as described in Figure 1A. Data are plotted relative to the mRNA levels in the SMI109 wt strain at pH 7, which was set to 1. Data from four biological replicates, each including two technical replicates, were used for the quantification. All data points included are shown as separate dots in the diagram. Statistical analysis was performed using the Mann–Whitney U-test (*P*< 0.0001, ****; *P*> 0.001, ***; *P*> 0.05, ns). (**B**) Effect of acid stress on SabA and AlpB protein in the SMI109 wt, Δ*hup*, and Δ*arsS*Δ*hup* strains, as monitored by immunoblot analysis. Normalized SabA expression levels (using expression levels of AlpB as reference) were calculated and plotted relative to the expression observed in SMI109 wt at pH 7, set to 1. The image above shows one representative immunoblot from the four independent experiments performed. All data points included are shown as separate dots in the diagram. Statistical analysis was performed using the Mann–Whitney U-test (*P*< 0.0001, ****; *P*> 0.05, ns). (**C**) The effect of acid stress on *hup* mRNA levels in SMI109 wt and Δ*arsS* strains as measured by RT-qPCR. Cultures were grown and analyzed as described in Figure 1A. The mRNA levels are plotted relative to the levels in SMI109 wt at pH 7, which was set to 1. Data from four biological replicates, each including two technical replicates, were used for the quantification. All data points included are shown as separate dots in the diagram. Statistical analysis was performed using the Mann–Whitney U-test (*P*< 0.0001, ****; *P*> 0.05, ns).

To analyze whether Hup also participates in the pH-dependent regulation of *sabA* expression in *H. pylori*, the Δ*hup* and Δ*arsS*Δ*hup* strains were constructed in strain SMI109 and exposed to acid stress. The effects on gene and protein expression were analyzed using RT-qPCR and immunoblot. The *sabA* mRNA levels and protein expression were clearly affected in the Δ*hup* strain after growing in broth, and expression was decreased by 2- to 3-fold (Figure 6A, B). This contrasted with our previously published results where we did not observe a change in *sabA* expression in the Δ*hup* strain (48). The main difference in the two studies is the growth conditions of the bacteria (agar versus broth). We analyzed samples from both growth conditions using the same reference genes as earlier and we got the same results. After growing on plate, the expression of *sabA* was almost 3-fold higher than after growing in broth and there was no change in *sabA* mRNA levels in the Δ*hup* strain, whereas in broth there was a 2-fold decrease in the Δ*hup* strain (Supplementary Figure S4B). When *sabA* mRNA and protein expression were analyzed in the Δ*hup* strain after shifting to acidic pH, expression was decreased even further, indicating that pH-dependent regulation still occurred in the absence of Hup in *H. pylori* (Figure 6A, B). Interestingly, when *sabA* mRNA and protein expression were analyzed in the Δ*arsS*Δ*hup* double mutant strain, the pH-dependent regulation of *sabA* expression was completely abolished (Figure 6A, B). We also observed that the increased *sabA* expression detected in the Δ*arsS* strain at pH 7 (Figure 1A, B) was reduced in the Δ*arsS*Δ*hup* strain, reverting the expression back to wt levels (Figure 6A, B). Simultaneously, the *ureA* mRNA levels were analyzed in the different strains by RT-qPCR. As previously described, the pH-dependent increase in *ureA* expression was abolished in both the Δ*hup* and Δ*arsS*Δ*hup* strains (Supplementary Figure S4C). Additionally, when the *hup* mRNA level was analyzed in wt and Δ*arsS* strains after acid stress, a clear upregulation of *hup* expression was observed in the wt strain at pH 5 (Figure 6C). This upregulation was not seen in the Δ*arsS* strain (Figure 6C). Taken together, these results suggest that both ArsR-P and Hup are important for the pH-dependent regulation of *sabA* expression in *H. pylori*. Expression of Hup is increased by acidic pH, and this potentiates ArsR-mediated pH-dependent regulation of *sabA* expression.

### ArsR-mediated pH-dependent regulation of *sabA* transcription occurs predominantly at BS II

**Figure 7.**
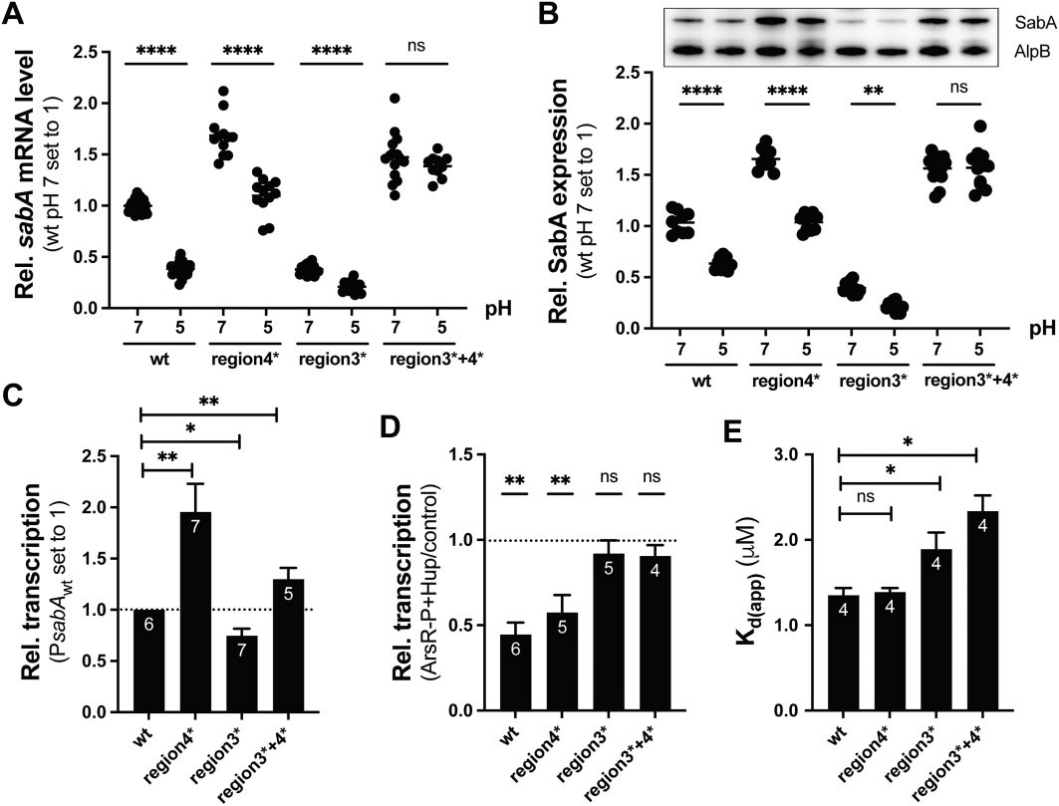
ArsR-mediated pH-dependent regulation of *sabA* occurs predominately via ArsR BS II. (**A**) The effect acid stress on *sabA* mRNA levels in SMI109 wt or isogeneic strains with scrambled region 3 (region 3*), region 4 (region 4*), or both (region 3* + 4*) as measured by RT-qPCR. Cultures were grown as described in Figure 1A. Data from four biological replicates, each including two technical replicates, were used for the quantification. All data points included are shown as separate dots in the diagram. Statistical analysis was performed using the Mann–Whitney U-test (*P*< 0.0001, ****; *P*> 0.05, ns). (**B**) Effect of acid stress on SabA and AlpB protein expression levels in SMI109 wt and isogeneic strains with scrambled DNA regions of the *sabA* promoter. From immunoblot analysis the normalized SabA expression levels (using AlpB expression levels as reference) were calculated and plotted relative to the expression observed in SMI109 wt at pH 7, which was set to 1. The image above shows one representative immunoblot from the four independent experiments. All data points included are shown as separate dots in the diagram. Statistical analysis was performed using the Mann–Whitney U-test (*P*< 0.0001, ****; *P*< 0.01, **; *P*> 0.05, ns). (**C**) IVT assay using P*sabA* templates with scrambled DNA sequences. A total of 0.5 nM template (P*sabA*_wt_; pAAG264, P*sabA*_region3*_; pAAG316, or P*sabA*_region4*_; pAAG267 and P*sabA*_region3*+4*_; pAAG334) was mixed with 10 nM of RNAP. Experimental procedures were performed as described in Figure 5G. The amount of transcript was quantified and plotted relative to the amount obtained with P*sabA*_wt_, set to 1. The bar graph represents the combined results from three independent experiments. The number of data points for each sample is written in the bar. Statistical analysis was performed on the raw data using the Mann–Whitney U-test (*P*< 0.01, **; *P*< 0.05, *). (**D**) IVT assay using His_6_-ArsR and P*sabA* templates with scrambled DNA sequences. A total of 0.5 nM template (P*sabA*_wt_; pAAG264, P*sabA*_region3*_; pAAG316, or P*sabA*_region4*_; pAAG267 and P*sabA*_region3*+4*_; pAAG334) was mixed with 10 nM of RNAP, 125 nM Hup, and 0 or 1 μM ArsR-P. Experimental procedures were performed as described in Figure 5B. The relative amount of transcript formed (ArsR-P/without ArsR-P) was calculated and plotted for each template DNA. The bar chart shows the combined results from at least two independent experiments. The number of data points for each reaction is written in the bars of the diagram. Statistical analysis was performed on the raw data using the Mann–Whitney U-test (*P*< 0.01, **; *P*> 0.05, ns). (**E**) Binding of His_6_-ArsR to P*sabA* with scrambled region 2–4 was analyzed by EMSA. A total of 7 nM TET-labelled P*sabA* DNA (407 bp PCR, 486/485-TET primers) with P*sabA*_wt_ (template pAAG264), P*sabA*_region3*_ (template pAAG316), P*sabA*_region4*_ (template pAAG267) or P*sabA*_region3*+4*_ (template pAAG334) was mixed with 0–4 μM ArsR-P. The amount of non-shifted DNA was quantified, and the amount of shifted DNA was calculated for each DNA at each concentration of ArsR-P used. From the amount of shifted DNA, calculations of *K*_d(app)_ for ArsR-P with each template DNA were performed for each experiment. The bar graph represents the results from two independent experiments, and the number of data points for each sample is written in the bars of the diagram. Statistical analysis was performed on the raw data using the Mann–Whitney U-test (*P*< 0.05, *; *P*> 0.05, ns).

The regulatory mode for OmpR-like regulators has been suggested to be dictated by the position of their binding site relative to the promoter, i.e. repression occurs at binding sites located downstream of the transcriptional start site (83). Because ArsR-P has a binding site on either side of the *sabA* core promoter, we asked if both sites are involved in ArsR-mediated pH-dependent regulation or only one of them. To analyze the impact of ArsR BS I on the pH-dependent repression of *sabA* expression in *H. pylori*, we introduced the scrambled region 4*, and to analyze the impact of ArsR BS II we introduced the scrambled region 3* into the SMI109 genome using the previously established counterselection method (48). We analyzed the *sabA* mRNA levels using RT-qPCR and SabA protein expression by immunoblot. We found that *sabA* expression was increased at pH 7 when DNA region 4 was scrambled and decreased when region 3 was scrambled (Figure 7A), still, there was clear pH-dependent reduction of *sabA* expression levels in both strains (Figure 7A). Similar results were obtained when SabA protein expression levels were analyzed (Figure 7B). Next, to analyze the impact of both binding sites, we introduced both scrambled regions of P*sabA* into the SMI109 genome and tested their effect on the pH-dependent repression of *sabA* expression in *H. pylori*. The increased *sabA* expression levels noted in the region 4* strain remained high in the double region 3* + 4* strain after growth at pH 7 (Figure 7A, B). Moreover, when the bacteria cultures were shifted to pH 5 the *sabA* mRNA and protein expression levels were not decreased, i.e. the pH-dependent repression was abolished in the region 3* + 4* strain (Figure 7A, B). As a control to ensure that the newly constructed strain was not affected in some more general feature of pH-dependent regulation, the expression levels of *ureA* mRNA were quantified by RT-qPCR in all strains and conditions used above. In keeping with the effect shown for the wt strain, there were a similarly increased *ureA* mRNA levels observed in all strains after shifting to pH 5 (Supplementary Figure S4D).

We next tested the impact of ArsR BS I and II on the ArsR-mediated repression of *sabA* transcription *in vitro*. Templates carrying DNA with the scrambled region 4* and region 3* were used in IVT assays together with P*sabA*_wt_. Firstly, we analyzed the basal transcription from P*sabA* with scrambled region 3* and 4* without Hup and ArsR-P and found that changing the long AT stretches in region 3* caused a decrease in the transcriptional output whereas changing the sequence of region 4* led to increased transcriptional output (Figure 7C). This corroborated what we observed in *H. pylori*, both at the mRNA and protein levels, as the region 3* strain showed 2-fold lower and region 4* strain a 2-fold higher *sabA* expression levels when compared to SMI109_wt_ (Figure 7A, B). This suggests that the sequences of both regions are important for optimal *sabA* transcription. Next, the amount of transcript was quantified after the addition of Hup and/or ArsR-P, and relative transcript levels with/without ArsR-P were calculated. We found that transcription of *sabA* was still repressed by ArsR-P when using a DNA template containing the scrambled region 4* (lacking BS I) but not when a template containing scrambled region 3* (lacking BS II) was used. When both regions were scrambled, no ArsR-mediated repression was observed (Figure 7D). This somewhat corroborates the results in *H. pylori*, where we still observed pH-dependent regulation of *sabA* expression when region 4* was scrambled in SMI109 but not when both regions were scrambled (Figure 7A, B). We know from our DNase footprint results that ArsR-P did not form a protected region at BS II and/or BS I when DNA with scrambled region 3* and/or 4* were used (Figure 3B–D). Furthermore, we obtained supporting results by analyzing binding of ArsR-P to P*sabA* by EMSA. We observed that the binding strength of ArsR-P was lower when region 3* was scrambled (*K*_d(app)_ of 1.89 ± 0.20 μM) compared to wt DNA (*K*_d(app)_ of 1.35 ± 0.09 μM) or region 4* DNA (*K*_d(app)_ of 1.39 ± 0.05 μM), and it was lowest when both region 3* and 4* were scrambled (*K*_d(app)_ of 2.34 ± 0.18 μM) (Figure 7E). Taking these results together, we suggest that binding of ArsR-P to BS II is the main regulatory DNA interaction for the pH-dependent repression of *sabA* expression. The residual pH-dependent regulation of *sabA* expression observed in the SMI109 region 3* strain is probably due to a yet unknown regulatory factor/mechanism occurring in *H. pylori* but not *in vitro*.

### ArsR-mediated regulation of *sabA* expression occurs by modulating the interaction between the α-subunit of RNAP and UP-like elements

The T-tract influences the spatial alignment of the P*sabA* core promoter and the A-boxes in the UP-like elements that are important for the interaction of the RNAP α-subunit (48), and thus affects transcriptional output. These UP-like elements were positioned at approximately −90 to −50 (proximal) and −130 to −105 (distal) and depending on the T-tract length and composition, interaction of the RNAP α-subunit is occurring at either site. Binding of RNAP to the core promoter was earlier estimated to occur at position −35 to +20 (48). ArsR BS I partly overlaps with the binding site of the RNAP σ-factor (core promoter), whereas ArsR BS II is positioned in the proximal UP-like element (Figure 3A). Interfering with any of these RNAP interactions by scrambling the DNA sequence affect the transcriptional output of *sabA*, both in *H. pylori* and *in vitro* (Figure 7A–C). We also found that scrambling the sequence affected the binding of ArsR-P to DNA (Figure 7E). We decided to use DNase I footprinting to determine if any of the ArsR binding sites would affect the interaction of RNAP with P*sabA* DNA or if ArsR-P would interact directly with RNAP to repress transcription. We used TET-labelled DNA and a longer, presumably more flexible, DNA fragment, compared to our earlier studies (48). Interaction of the RNAP α-subunit with the longer DNA (407 bp) was only observed with the distal UP-like element (position −124 to −92), and binding of the RNAP σ-factor on the core promotor was at position −17 to +15 (Supplementary Figure S5A). Using the shorter DNA (298 bp), like in our earlier analyses (48), the RNAP interacted with the core promoter at the same position (−17 to +15) as with long DNA but only with the proximal UP-like element (−87 to −65). First, we mixed RNAP, Hup and/or ArsR-P with P*sabA* DNA from SMI109 and analyzed changes in the binding pattern of RNAP and ArsR-P using DNase I footprint analysis (Supplementary Figure S5B). While the binding of ArsR-P to BS II and the binding of RNAP to the distal UP-like element remained distinguishable, the binding of ArsR-P to BS I and RNAP to core promoter merged. In the presence of ArsR-P, an extended protected region was observed from −17 to +20, corresponding to binding site of both RNAP and ArsR-P BS I. With both ArsR-P and Hup present, the footprint extended further up to +27 (Supplementary Figure S5B). Given the substantial difference in the binding strength between RNAP and ArsR-P, we conducted the subsequent footprint assays separately.

**Figure 8.**
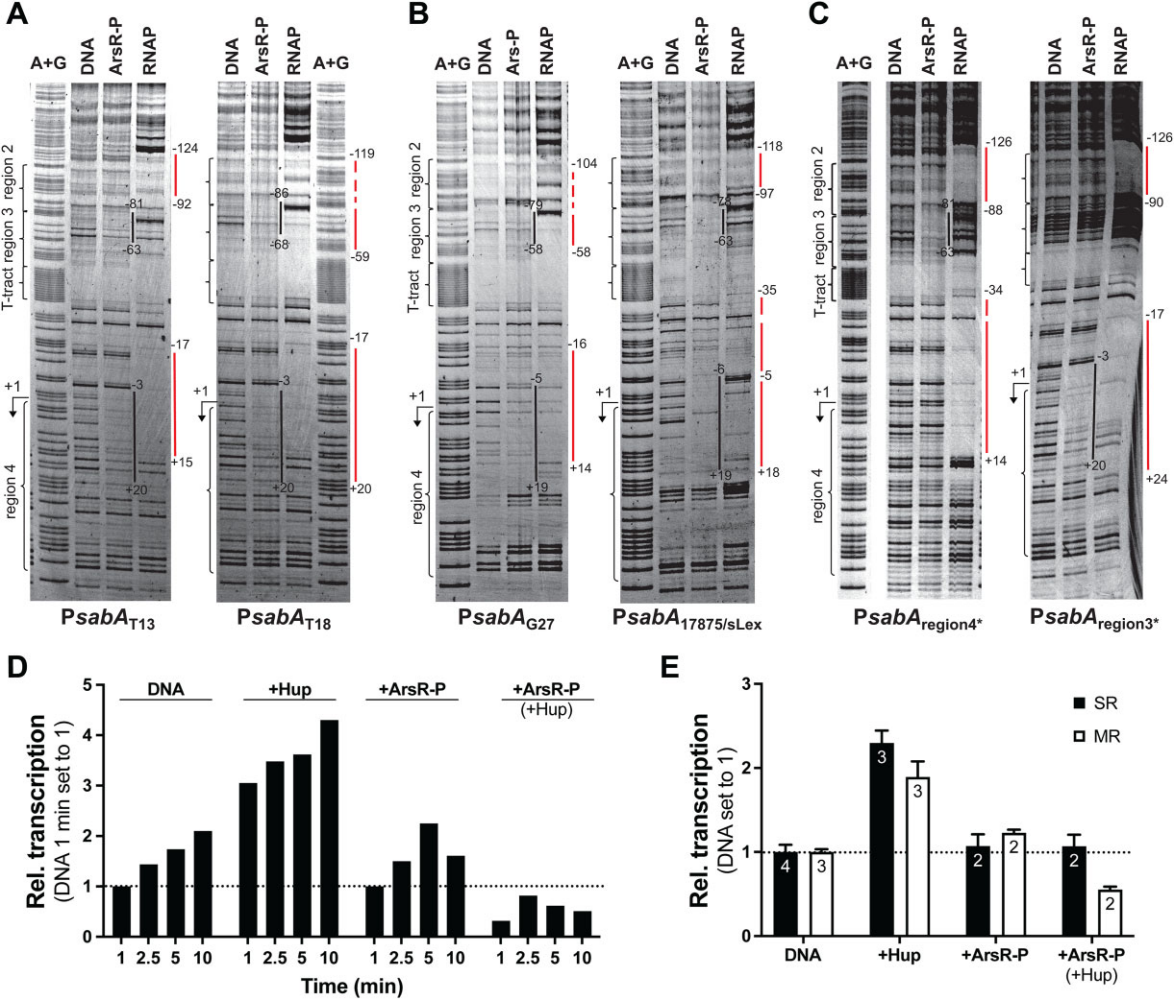
Binding of ArsR to BS II of P*sabA* DNA occurs near the binding site of the RNAP alpha subunit. (**A–C**) DNase I footprint analysis of His_6_-ArsR and RNAP binding to: (A) P*sabA* SMI109 DNA with different T-tract lengths (T_13_ and T_18_); (B) P*sabA* from different *H. pylori* strains (G27 and 17875sLex); (C) P*sabA* with scrambled DNA (region 3* or 4*). A total of 25 nM TET-labelled DNA (407–412 bp PCR 486/485-TET primers) was mixed with protein storage buffer (lane DNA), 10 μM ArsR-P (lane ArsR-P), or 300 nM *E. coli* σ^70^-RNAP (lane RNAP). The template plasmids used to generate the TET-labelled DNA were the same as the IVT plasmids used in Figures 5G and 7C. The Maxam and Gilbert DNA sequencing reaction (lane A + G) shows the sequences of DNA used. Binding sites are marked by solid lines (black for ArsR-P, red for RNAP), and the nucleotide positions of the binding sites are shown along the right side. Transcriptional start site (+1), T-tract, and the regions described in Figure 3A are indicated along the left side. The images show one representative gel of at least two independent experiments. The line representation of the protection pattern for ArsR-P and RNAP on P*sabA* is found in Supplementary Figure S6. (**D**) Time-course IVT assay with P*sabA* from SMI109. A total of 0.5 nM template (pAAG264) and 10 nM RNAP was used in multiple-round transcriptions run at 37°C together with 125 nM Hup or 1 μM ArsR-P. Time was recorded after the addition of nucleosides, and samples were removed for analysis after 1, 2.5, 5 and 10 min. The amount of transcript formed was quantified from the gel image. The transcription level without additional protein (DNA) at *T* = 1 min was set to 1, and the amount of transcript formed in the presence of only Hup or ArsR-P was plotted relative to that. The amount of transcript formed after the addition of both Hup and ArsR-P was plotted relative to the amount of transcript formed with only Hup at *T* = 1 min, which was set to 1. (**E**) Single and multiple-round IVT assays with P*sabA* from SMI109. A total of 0.5 nM template (pAAG264) and 10 nM of RNAP together with 125 nM Hup or 1 μM ArsR-P, was used in single (SR) or multiple round (MR) transcriptions run at 37°C. The amount of transcript formed after 10 min was quantified from the gel image. The transcription level without additional protein (DNA) was set to 1, and the amount of transcript formed in the presence of only Hup or ArsR-P was plotted relative to that for each assay. The amount of transcript formed after the addition of both Hup and ArsR-P was plotted relative to the amount of transcript formed with only Hup, which was set to 1. The bar graph shows the results from two independent experiments, and the number of data points for each reaction is written in the bars of the diagram.

Next we decided to analyze if the T-tract length would affect the interaction between ArsR-P and RNAP. In the P*sabA* SMI109 T_13_ DNA, the RNAP binding region at the core promoter partly overlapped with ArsR-P BS I, but not with BS II. The interaction of the RNAP α-subunit occurred at −124 to −92 while the σ-subunit bound at −17 to +15 (Figure 8A, left and Supplementary Figure S6). However, in the case of the P*sabA* SMI109 T_18_ DNA, the RNAP binding overlapped with both ArsR-P binding sites. Here the interaction of the RNAP α-subunit and σ-subunit occurred at −119 to −59 and −17 to +20, respectively (Figure 8A, right and Supplementary Figure S6). G27 and 17875/sLex have, in addition to small sequence variations in the *sabA* promoter region, different lengths of their T-tracts (Figure 3A). Similar results as for the T-variants were observed when the binding of RNAP and ArsR-P to P*sabA* DNA from strains G27 (Figure 8B, left) and 17875/sLex (Figure 8B, right) was analyzed. For the *sabA* low-expressing strain G27, the RNAP and ArsR-P binding sites overlapped at both positions −104 to −58 and −16 to +14, just as in the T_18_ DNA (Supplementary Figure S6). In contrast, with DNA obtained from the *sabA* high-expressing strain 17875/sLex, the interaction of RNAP occurred at approximately the same positions as for T_13_ DNA, namely −118 to −97 and −35 to +18 (Supplementary Figure S6). The RNAP binding at the core promoter was extended a bit upstream compared to T_13_ DNA (Figure 8A, B). This likely explains why the pH-dependent regulation was absent in the *sabA* low-expressing strains, i.e. because of the changed interaction of the RNAP α-subunit to UP-like elements in P*sabA* DNA, thus competing with the binding of ArsR-P at BS II, and the shift in the interaction of RNAP with the core promoter, thus competing with the binding of ArsR-P at BS I (Supplementary Figure S6).

Transcription initiation is a complex multistep process involving promoter recognition, open-complex formation and/or stability, and promoter escape (84,85 and references therein). To try and determine at which step in the transcriptional initiation process Hup and ArsR-P affect *sabA* transcription, a time-course IVT experiment was conducted. The amount of transcript formed was analyzed from 1 to 10 min after the addition of nucleosides (Figure 8D). About a 2-fold increase in *sabA* transcript was formed over this time frame when only DNA and RNAP was present. When Hup was included, a corresponding transcript level was reached already within 1 min, and it continued to increase over time. ArsR-P alone did not appear to change the amount of transcript formed at any time point, again suggesting that ArsR-P did not affect *sabA* transcription in the absence of Hup *in vitro*. As Hup and ArsR-P were both present with DNA and RNAP, a clear decrease in the transcript level was observed as compared to when Hup was present alone. The repression was evident already within 1 min and continued until 10 min had passed (Figure 8D). To further investigate the mechanism of action through which Hup and ArsR-P affect *sabA* transcription, we compared the amount of transcript formed in single-round versus multiple-round IVT assays. In the single-round assay (SR), due to the presence of heparin the RNAP is only allowed to transcribe once from each template, whereas in the multiple-round assay (MR), re-binding of RNAP and multiple initiations of transcriptional cycles will occur until the reaction is stopped. These assays were run with or without Hup and/or ArsR-P. Interestingly, transcription from the *sabA* promoter was equally stimulated by Hup in the two assays, but repression by ArsR-P (in the presence of Hup) was only observed in the multiple round assay (Figure 8E). Again, no effect was observed with ArsR-P alone. These results suggest that Hup and ArsR-P affect the initial steps in the transcription process. As anticipated for a nucleoid-associated protein with a role in global DNA topology, it appears that Hup is influencing the transcriptional process from the very onset and thereby preceding the action of ArsR-P.

**Figure 9.**
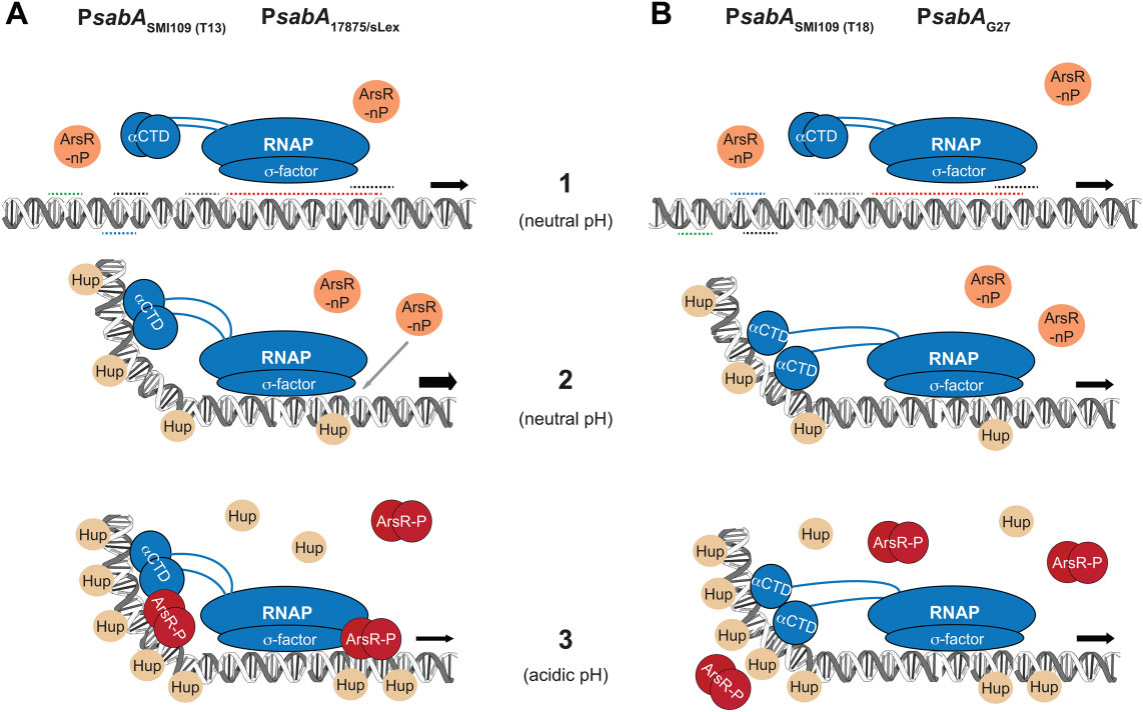
Model for ArsR-mediated pH-dependent regulation of *sabA* expression in *H. pylori*. Schematic overview how regulation of *sabA* expression occurs via the repressing activity of phosphorylated ArsR (ArsR-P) and the DNA interaction by Hup in *H. pylori*. (**A**) A genetic variant with P*sabA* DNA that generates *sabA* high-expression (optimal T-tract length, SMI109 (T_13_) or 17875/sLex). (**B**) A genetic variant with P*sabA* DNA that generates *sabA* low-expression (suboptimal T-tract length, SMI109 (T_18_) or G27). The grey dotted line shows the position of the T-tract, the black dotted lines show the ArsR binding sites, the red dotted line shows the binding site of sigma-subunit (σ^70^) of the RNA polymerase (RNAP) and green or blue dotted lines show distal or proximal UP-like elements, binding site for the alpha-subunit C-terminal domains (αCTDs) of RNAP. Horizontal arrows ahead of the RNAP indicate relative expression levels. Regulatory interactions and expressions of the two genetic variants are illustrated in scenarios (1-3) of different pH conditions. **1**) Transcription from a non-curved *sabA* promoter is very low as the RNAP αCTDs interaction with UP-like elements is not ideal due to poor DNA bending in the absence of Hup (A1) or a change in axial alignment due to T-tract length (B1). **2**) At pH-neutral conditions, Hup together with an optimal T-tract length will mediate the correct curvature and spatial alignment needed for the optimal positioning of RNAP at the core promoter and thus increase transcription from the *sabA* promoter (A2). When a suboptimal T-tract length distorts the interaction of αCTDs to the UP-like elements, the positioning of RNAP at the core promoter will be changed, resulting in decreased transcription (B2). **3**) Exposure to acidic pH, increase Hup expression, and ArsR becomes phosphorylated. Additional Hup will bind to P*sabA* DNA to change the DNA curvature by narrowing the size of the minor groove. This aid binding of ArsR-P to the minor groove of BS I and II (A3). The reduced change in DNA curvature with suboptimal T-tract length reduces the binding strength of ArsR-P to P*sabA* DNA (B3). Binding of ArsR-P to P*sabA* DNA will interact directly with the RNAP αCTDs at BS II or will change the interaction of αCTD with the UP-like element, and this will result in the repression of transcription from P*sabA*. Because binding of the RNAP σ^70^-subunit at the core promoter is very strong and partly overlaps with BS I, most of the ArsR-mediated repression occurs via BS II. However, depending on the positioning of RNAP, there might be a possibility for ArsR-P and/or ArsR-nP to bind to BS I, further repressing transcription by acting as a roadblock to RNAP (A3). For the *sabA* low-expressing strains (with suboptimal T-tract length), the binding of the two αCTDs at both the distal and proximal UP-like elements, in combination with the shifted σ^70^-subunit binding at the core promoter, overlaps with ArsR BS II and BS I. This results in displacement of ArsR-P at both binding sites, and no ArsR-mediated repression of transcription will occur (B3).

We found changes in *sabA* transcript levels with both the scrambled region 3* and region 4* DNA templates, and this also affected the pH-dependent *sabA* expression in *H. pylori* and ArsR-mediated repression *in vitro* (Figure 7A, B and D). To determine if these differences in transcript levels would be due to differences in the interaction of RNAP with the scrambled DNA, a DNase I footprint analysis was performed. When region 4* was scrambled, no apparent difference in the RNAP binding pattern was observed except that the core promoter extended to -34 to + 14, which was further upstream compared to the binding pattern on P*sabA*_wt_ DNA (Figure 8C, left, and Supplementary Figure S6). Similarly, the binding of RNAP to the core promoter in 17875/sLex DNA was also shifted towards the upstream DNA sequences (Figure 8B, right), and these DNAs had a much higher transcriptional output compared to P*sabA* from SMI109 (Figures 5G and 7C). Unexpectedly, when binding of RNAP to P*sabA* with scrambled region 3* was analyzed, a shift in the core promoter binding site more downstream to −17 to +24 was observed, covering the entire ArsR BS I (Figure 8C, right, and Supplementary Figure S6). Our results suggest that those sequence changes in the proximal UP-like element, and thereby affecting the interaction by αCTD, would somehow alter the positioning of the RNAP σ-factor at the core promoter (Figures 7A–C and 8C). This could also explain why there was no repression of ArsR-P on region 3* DNA *in vitro* (Figure 7D) as an extended RNAP binding at the core promoter would not allow ArsR-P to bind to any part of BS I. It should be noted that no binding to BS II of ArsR-P occurred with this DNA (Figure 3C). Similarly, changes in the length of the T-tract that affect the axial alignment between UP-like elements and the core promoter evidently resulted in a similar scenario (Figures 4C-D, 5G and 8A-B). Overall, the rate-limiting step for optimal *sabA* transcription seems to be the interaction of the RNAP α-subunit to UP-like elements leading to the correct positioning of RNAP at the core promoter, likely resulting in faster formation and/or more stable open complexes. Increased DNA bending by Hup would be stabilizing this interaction and thereby increasing the transcriptional output. ArsR-P binding at BS II could disrupt the interaction by the α-subunit of RNAP with UP-like elements and/or the DNA wrapping needed for correct positioning of RNAP, thus resulting in repression of transcription as illustrated in the schematic models (Figure 9).

## Discussion

The acid stress response in *H. pylori* is an intricate process, and the acid-responsive ArsRS regulon has been the focus of a series of studies, but the mechanistic details behind transcription regulation have not been fully elucidated. The fact that *arsR* is essential for bacterial growth has complicated the ability to molecularly dissect the role of ArsR in gene regulation in *H. pylori*. To reveal the molecular details of ArsR-mediated pH-dependent regulation of the SabA adhesin, we analyzed protein and mRNA levels in *H. pylori* using a series of deletion mutants in combination with multiple direct DNA binding assays and IVT and drew the following conclusions: (i) ArsR-P represses *sabA* transcription by binding directly to two binding sites (BS I and BS II) in the *sabA* promoter (Figures 2C and 5B). ArsR binds to P*sabA* DNA independently of its phosphorylation status (Figure 2A, B), but ArsR-nP binds weakly to BS I while ArsR-P binds strongly to both BS I and BS II (Figure 2D). ArsR-P shows higher binding strength to BS II, and the repressing effect on *sabA* transcription occurs predominantly via binding to this site (Figures 2D and 7E). (ii) DNA topology is important for ArsR binding (Figure 3B–D), and upon phosphorylation ArsR shifts binding from the major to minor groove of the DNA (Figure 5C). The repressing effect of ArsR-P is depleted *in vitro* when DNA containing BS II (region 3*) is scrambled to reduce AT stretches (Figure 7D) or in the absence of Hup (Figure 5B and H). (iii) pH-dependent regulation is abolished in *sabA* low-expressing strains (G27 and the T_18_ variant) (Figure 4C, D), a strain where both ArsR-P BS I and BS II are scrambled (Figure 7A, B), and in a strain depleted of both *hup* and *arsS* (Figure 6A, B). (iv) ArsR-P affects multiple interactions between RNAP and P*sabA* DNA, thereby repressing transcription (Figure 8A–C). (v) Hup increases *sabA* transcription by interacting with AT-rich DNA regions containing both ArsR binding sites (Figure 5E, F). Hup expression itself is increased at acidic pH and is regulated by the ArsRS system (Figure 6C). This supports a model where pH-dependent repression of *sabA* transcription is mediated mainly via interactions between ArsR-P and the proximal UP-like element. This in turn, with the help of the DNA-bending protein Hup, affects the interaction of the RNAP α-subunit with the distal UP-like element and/or the communication between the RNAP α- and σ-subunits, thus resulting in changes in the transcriptional output from the *sabA* promoter. Moreover, binding of ArsR-nP or ArsR-P to BS I acts as a roadblock to slow down the progression of RNAP from the core promoter and thus decreases the transcriptional output even further (Figure 9).

### DNA topology and DNA structuring proteins play important roles in pH-dependent gene regulation

Most transcriptional regulators bind to specific nucleotide base patterns in the major groove, although some can distinguish molecular elements presented by the minor groove of DNA. The ArsR protein, belonging to the family of OmpR-like regulators, has its activity allosterically controlled by phosphorylation and its binding is influenced by DNA topology (69,70). Our results demonstrate that ArsR recognizes intrinsic changes in DNA topology, mediated by long AT stretches (>4 As or Ts) in the P*sabA* DNA, as well as the T-tract (Figure 3A). When AT stretches in region 3 and 4 were scrambled, it led to the abolishment of ArsR-P binding (Figure 3B–D) and the ArsR-mediated repression of *sabA* transcription (Figure 7A, B and D). Additionally, altering the length of the T-tract from T_13_ to T_18_ resulted in a decrease the binding strength of ArsR-P to P*sabA* DNA (Figure 4A, B). Interestingly, our findings reveal that ArsR binds to P*sabA* DNA independently of phosphorylation (Figure 2A), while ArsR-nP binds exclusively to BS I, whereas ArsR-P binds to both BS I and BS II (Figure 2C, D). This suggest that there may be a difference in the sequence specificity or binding off-rate between ArsR-nP and ArsR-P. Here, we also demonstrate that balanced binding of ArsR-P occurs on both DNA strands (Figure 2C), a feature that is associated with high specificity (86). Long AT stretches of DNA lead to intrinsic bends (71) and narrowing of the DNA minor groove. Consequently, the electrostatic potential creates specific binding sites for positively charged amino acids, primarily arginines (87), and it has been suggested that two amino acids in the DNA binding domain of ArsR - K190 and R217 - are involved in binding (88). Upon phosphorylation, ArsR binding shifts from the major to minor groove, thus shifts from recognizing sequences to recognizing structural determinants (Figure 5C). This shift in binding mode is clearly a critical determinant of binding strength and transcriptional readout (Figures 2C and 5B), as ArsR binds to DNA phosphorylation-independently (Figure 2A). A possible explanation for this might be that binding of ArsR-nP to the major groove potentiates the binding of ArsR-P to the minor groove. A similar type of shift in groove binding mode has been described for the *H. pylori* Fur protein in FeON-FeOFF regulation (89). HP1043, another response regulator in *H. pylori*, has been shown to interact with both the major and minor groove in a sequence-specific manner. The HP1043 acts, in contrast to ArsR, only in a phosphorylation-independent manner (90,91).

In addition to ArsR-P, pH-dependent regulation of *sabA* transcription was clearly affected by the DNA-structuring protein Hup. The role of Hup in pH-dependent regulation in *H. pylori* has been addressed in two previous studies (77,78). Under acidic conditions, the levels of *hup* mRNA (Figure 6C and ref 92), as well as the stability of the Hup protein, increase (93). However, in contrast to our *in vitro* results, in the *hup* mutant strain the pH-dependent regulation of *sabA* expression was not completely abolished. For other bacterial species, if a nucleoid-associated protein is missing it can be compensated for by others (74,94). In many bacterial species, two homologues of Hup subunits are encoded, and these can form homo- and heterodimers before binding to DNA. In *H. pylori* there is only one *hup* gene (75), however, it seems as if at least one more Hup-like DNA-binding protein is present in *H. pylori*, namely HP0119 (76). The expression of *hp0119* is also increased at acidic pH and is regulated by ArsRS (19,66). In the absence of Hup, HP0119 might take over the role as the DNA-structuring protein and thereby ensure that ArsR-P acts as a repressor of *sabA* expression. Being a global transcriptional regulator, lack of Hup most certainly affects additional factors that might assist ArsR-P in pH-dependent gene regulation in *H. pylori*. This illustrates the difficulty of studying the mechanistic effects on gene expression of mutants of global regulators and why it is a necessity to combine *in vivo* observations with *in vitro* experiments.

### ArsR mediates transcriptional repression by modulating the interaction between the α-subunit of RNAP and UP-like elements

Remarkably, we show that ArsR-P effectively represses transcription by binding mainly upstream of the transcriptional start site (BS II), but also downstream (BS I) in the *sabA* promoter region. This is rather surprising because the regulatory mode of OmpR-like regulators has been proposed to be dictated by the position of its binding site relative to the promoter (83). With the dual binding sites and knowing that response regulators bind DNA as dimers, a model of a repressosome formed by DNA looping was tempting at first (95,96). This would trap the RNAP, inhibiting transcription elongation and/or preventing binding of RNAP to the core promoter. Several studies have shown that DNA looping requires DNA-structuring proteins like Hup (97–99) and that it is also dependent on upstream promoter sequences (100,101). However, when the two binding sites were separately scrambled no effect on ArsR-P binding to the other site was observed (Figure 3B–D). We did see a change in ArsR binding strength to DNA when BS II was scrambled (Figure 7E) and a lack of repression by ArsR-P on *sabA* transcription *in vitro* (Figure 7D), but not when BS I was scrambled (Figure 7D, E). Both sites needed to be scrambled before pH-dependent repression was lost in *H. pylori* (Figure 7A, B). These results led us rule out the repressosome model.

We instead suggest that ArsR-P binding to BS II is the predominant reason for the repression of *sabA* transcription. This binding site is located adjacent to the binding site of the C-terminus domains of the two RNAP alpha subunits (αCTDs). These subunits bind in tandem to positions in the promoter DNA known as the UP-element (102,103). Due to the flexible linker, the αCTDs can move about 30 bp along the DNA (104,105), and depending on the DNA structure this might be extended even further (106). αCTDs recognize intrinsic bends and interact with the minor groove side of the DNA helix (107). We suggest that the interaction of αCTD to UP-like elements is the rate-limiting step in the transcriptional regulation of *sabA* (Figure 7A–C), and this affects the positioning of the σ-factor at the core promoter and thus affects transcription initiation (Figure 8A–C and Supplementary Figure S6). ArsR-P BS II is located between the αCTDs and the σ-factor binding sites of RNAP, and because ArsR-P interacts with the minor groove of the DNA this can result in distortion of the DNA structure in the immediate vicinity, thus affecting the direct interaction of αCTDs with DNA. An alternative scenario is that ArsR-P affects the protein-protein interaction between αCTDs and σ-factor, as described for the *lacUV5* promoter in *E. coli* (108).

The *H. pylori* RNAP is not identical to the *E. coli* RNAP since its β and β’ subunits are expressed as a fusion protein (109) and because the αCTD has an additional 5^th^ helix (110). However, the binding of the αCTDs to UP-like elements is conserved (110). Nevertheless, both we and others have shown that *E. coli* RNAP works well at transcribing *H. pylori* promoters (48,90,111). We noticed a difference in the ArsR-mediated pH-dependent repression of *sabA* transcription when BS II was scrambled *in vitro* (Figure 7D) as compared to the repression seen in *H. pylori* (region 3* strain) (Figure 7A, B) in which repression was not lost until both binding sites were scrambled. This might be due to the fact that in the absence of a functional BS II, ArsR-P instead binds to BS I and thus acts as a roadblock if the RNAP σ-factor is positioned in such a way that it is not covering that binding site in a similar manner to what we observed *in vitro* (Figure 8A-C). It could also be that in our region 3* strain, an alternative ArsR binding site appears further upstream, due to the longer and more flexible DNA structure, as we observed *in vitro* when region 2* was scrambled in P*sabA* DNA (Figure 3C). It also could be that the extra helix in the *H. pylori* αCTD allows for multiple-factor interactions that are necessary for further stabilization of the RNAP binding position and thus contributes to pH-dependent repression in *H. pylori*. Similar to what was seen for the region 3* *H. pylori* strain, a strain lacking the sensor kinase ArsS could retained the pH-dependent regulation of *sabA* expression. This is something that has been discussed earlier and has been observed for other genes (62). An alternative scenario is that ArsR is phosphorylated by one of the other sensor kinases or that ArsR-nP binding to BS I can help to repress transcription in the absence of ArsR-P.

### Stochastic events add an additional layer for fine-tuning of pH-dependent regulation in *H. pylori*

We have previously demonstrated that the interaction of the RNAP αCTDs with P*sabA* DNA is affected by the T-tract length, resulting in changes to the transcriptional output (48). Here we show that the T-tract length also affects the ArsR-mediated pH-dependent regulation of *sabA* expression (Figures 4D and 5H). The lack of a complete mismatch repair system and the deficient proofreading activity in DNA polymerase I together with natural competence for transformation (112,113) causes exceptionally high genetic diversity and variability in *H. pylori* (68,114,115). Genes coding for essential functions, such as *ureA* and *arsR*, are often well-conserved, while genes encoding surface-exposed proteins exhibit higher variability (116,117). The wealth of SSRs in *H. pylori* constitute hotspots for slipped-strand mispairing, which rapidly creates a large pool of heterogenous clones (118–121). The high genetic diversity within *H. pylori* populations found in biopsy materials isolated from the corpus and from the less acidic antrum region of infected stomachs (33,48,122,123) clearly demonstrates their ability to adapt to different acidic conditions. Mutations in genes encoding outer membrane proteins and proteins involved in chemotaxis, motility, toxin production, and regulatory functions are over-represented among niche-specific mutations (123).

Here, we show that the clonal variation in the T-tract length of the *sabA* promoter affected binding strength of ArsR-P to P*sabA* DNA *in vitro* (Figure 4A, B), and thus affected ArsR-mediated pH-dependent regulation in *H. pylori* (Figure 4C, D). In the *sabA* low-expressing strains, which are devoid of pH-dependent regulation, ArsR BS II overlaps with the binding site of the RNAP α-subunit, and because RNAP binds with higher affinity than ArsR-P to P*sabA* it is likely that RNAP out-competes the binding of ArsR-P to impede repression in these strains (Figure 8A, B and Supplementary Figure S6). Our study explains mechanistically how the variation in the length of the T-tract in P*sabA* affects the DNA topology, the binding of ArsR-P, and the interactions with RNAP and thus affects the transcriptional output in response to variable pH. Other recent studies have also addressed the role of SSRs for fine-tuning of gene expression, such as the G-tract in the 5′UTR of the *tlpB* mRNA, which affects gene expression of *tlpB* post-transcriptionally (51), as well as differences in the T-tract length located in the sRNA *nikS* promoter, which affects its expression (49,50). The *sabA* promoter is not unique in having long T- or A-tracts just upstream of the -35 promoter element and in being repressed by ArsS and/or pH. Several genes, many of them encoding other outer membrane proteins, such as the genes *hopD*, *hofA*, *hopA*, *sabB* and *hopQ*, have these regulatory elements in their promoter regions, and they are positioned in a similar way as for *sabA* (10–12,22,24–26). This implies that pH-dependent regulation of these genes is likely to be mediated by the same mechanism as for *sabA*.

With this study, we have contributed insights into the molecular properties of ArsR and the properties of the promoter DNA targets for ArsR-mediated pH-dependent regulation. We have shown that DNA topology is crucial for ArsR binding to DNA, where the long AT stretches sculpt the DNA and direct the binding of ArsR-P. We suggest that ArsR-mediated pH regulation of *sabA* mainly occurs by affecting interactions with the RNAP α-subunit to promoter DNA with the help of Hup. With the AT-rich *H. pylori* genomic DNA, it is likely that Hup-like proteins bind to many positions. This binding to the minor groove will allosterically improve the interaction of other regulatory factors as well as RNAP. Under acidic conditions, the increased expression of these regulators is likely to both protect the DNA from damage and redirect gene expression by changing the DNA topology. Clearly, the numerous SSRs in *H. pylori* and the stochastic genetic variation that affects the length of SSRs constitute important regulatory elements for fine-tuning gene expression and facilitating host adaptation to different environmental stresses.

## Supplementary Material

gkae188_Supplemental_File

## Data Availability

The data underlying this article will be shared on reasonable request to the corresponding author.

## Notes

Present address: Pär Gideonsson, Department of Medical Biosciences, Umeå University, SE-90187 Umeå, Sweden.

Present address: Abhayprasad Bhat, Department of Microbiology, Tumor and Cell Biology, Karolinska Institute, SE-17177 Stockholm, Sweden.
