# Supplementary material for: Molecular insights into the fine-tuning of pH-dependent ArsR-mediated regulation of the SabA adhesin in Helicobacter pylori
Source: Nucleic Acids Res. 2024 Mar 18;52(10):5572–95. doi: 10.1093/nar/gkae188 (PMC11162790; doi:10.1093/nar/gkae188)
Supplement: gkae188_Supplemental_File [file gkae188_supplemental_file.pdf]

Supplementary data

Molecular insights into the fine-tuning of pH-dependent ArsR-mediated regulation of the SabA adhesin in *Helicobacter pylori*

Anna Åberg, Pär Gideonsson[†], Abhayprasad Bhat[†], Prachetash Gosh and Anna Arnqvist*

Content

1. **Supplementary Tables S1-S3**
2. **Supplementary Figures S1-S6 and Figure legends**
3. **References for Supplement**

Table S1. Strains and plasmids used in this study

Name	Characteristics	Reference
Strains		
DH5α	*E. coli*	Laboratory stock
BL21 (DE) /pLYS-S	*E. coli*, F- *ompT hsdSB gal dcm* (T7 polymerase), pLysS (T7Lysozyme, Cm^R), used for protein purification, Cm^R	Novagen
G27	*H. pylori* clinical isolate, Italy, Gastritis	(1)
17875/sLex	*H. pylori* clinical isolate, Australia, Gastritis	(2)
SMI109	*H. pylori* clinical isolate, Sweden, Gastric cancer	(3)
SMI109P*sabA*::*lacZ*	SMI109 harboring pAAG129 on the chromosome, Km^R	This study
SMI109Δ*arsS*	Δ*arsS*::*cat* variant of SMI109, Cm^R	This study
SMI109Δ*sabA*::*rpsLCAT*	*sabA* promoter in SMI109 replaced by *rpsLCAT* cassette, Cm^R	(4)
SMI109 T-variants	Constructed variants of SMI109 with different repeat tract length in *sabA* promoter; T_{13} and T_{18}	(4)
SMI109 scrambled P*sabA* region4*	Region 4 (+2 to +38) of *sabA* promoter in SMI109 scrambled to reduce AT-stretches	This study
SMI109 scrambled P*sabA* region3*	Region 3 (-77 to -48) of *sabA* promoter in SMI109 scrambled to reduce AT-stretches	This study
SMI109 scrambled P*sabA* region3* and region4*	Region 3 (-77 to -48) and 4 (+2 to +38) of *sabA* promoter in SMI109 scrambled to reduce /T-stretches	This study
SMI109Δ*hup*	Δ*hup*::*aphA-3* variant of SMI109, Km^R	(4)
SMI109Δ*arsS* Δ*hup*	Δ*arsS*::*cat,* Δ*hup*::*aphA-3* variant of SMI109, Cm^R Km^R	This study
Plasmids		
pUC19	General cloning vector, Cb^R	(5)
pAAG179	Δ*arsS::rpsLcat* construct cloned in pUC19, Cm^R, Cb^R	This study
pKD4	Template plasmid for frt-flanked *aphA-3* cassette, Km^R	(6)
pBW	*lacZ* promoter fusion vector for *H. pylori*, Km^R	(7)
pCR TOPO	General cloning vector, Cb^R	Life Technologies
pAAG129	*sabA* promoter from SMI109 cloned in pBW, Km^R	This study
pCR+$ArsR_{109}$	*arsR*$_{WT}$ from SMI109 cloned in pCR TOPO, Cb^R	This study
pET28a	N-terminal His_6-tag, T7 promoter, Km^R	Novagen
pAAG156	*arsR*$_{WT}$ from SMI109 cloned in pET28a, Km^R	This study
pET21a	C-terminal His_6-tag, T7 promoter, Km^R	Novagen
pAAG190	*hup* from SMI109 cloned in pET21a, Cb^R	This study
pTE103	*In vitro* transcription vector, Cb^R	(8)
pAAG261	*ureA* promoter (P*ureA*, -250 to +87) from SMI109 cloned in pTE103, Cb^R	This study
pAAG264	*sabA* promoter (P*sabA*, -245 to +74) from SMI109 cloned in pTE103, Cb^R	This study
pAAG265	P*sabA* (-248 to +74) from G27 cloned in pTE103, Cb^R	This study
pAAG266	P*sabA* (-246 to +74) from 17875/sLex cloned in pTE103, Cb^R	This study
pAAG267	P*sabA* with scrambled region 4* from SMI109 cloned in pTE103, Cb^R	This study
pAAG286	P*sabA* from SMI109 T_{18} cloned in pTE103, Cb^R	This study
pAAG315	P*sabA* with scrambled region 2* from SMI109 cloned in pTE103, Cb^R	This study
pAAG316	P*sabA* with scrambled region 3* from SMI109 cloned in pTE103, Cb^R	This study
pAAG328	*sabA* ORF (+205 to +425) from SMI109 cloned in pTE103, Cb^R	This study
pAAG334	P*sabA* with scrambled region 3*+4* from SMI109 cloned in pTE103, Cb^R	This study

Table S2. Oligonucleotides used in this study

Name	Sequence (5' to 3')
**Plasmid constructions**	
ureA-1	ATGAATTCTAGAGCACTACCTTGACATGG
ureA-2	ATGGATCCCTTATCTAACTCTTTTGGGGT
sabA-1	ATGAATTCCTCTAGCAATGTGTGG
sabA-3	ATGGATCCCTTTTTCATAAAAGTGTTCCTT
P173	ACTATGACTATGTTGTCAGTTCACATTCTCATGATCAGAACCTAATTTTACTATAA
P174	TGATCATGAGAATGTGAACTGACAACATAGTCATAGTCAAAAACATTACTTTAAGGAACA
P181	GTCGTTAACACAACATACGCATTCGTGGTCACTAAGAAAGATAAATAGGTTAAAATACTCC
P182	CTTAGTGACCACGAATGCGTATGTTGTGTTAACGACGAATTTTATCATCAGTATTTAATG
P183	TCTTGAGCTGCTACAGCTGGTTAGATTCTGATTTTTTTTTTTTTTGGAAATCCAATAAATT
P184	TCAGAATCTAACCAGCTGTAGCAGCTCAAGAGAAGCGTTGATTTTTGTAACGATAAA
sabA-qP1F	TGAACGAAAAATACGAGCAA
sabA-qP1R	CCCGCATAAAGACTCCAAAA
arsR-1	GCACATATGGAAGTTTTAATGATAGAAG
arsR-2	GCAGGATCCTCAGTATTCTAATTTAT
hup-6	GTCCATATGAACAAAGCGGAATTTATTG
hup-7	CATCTCGAGCTTGCCTTCTTCAAC
**Deletion/ insertion mutants in *H. pylori***	
P68	CATTTCATGGCGTTTGGTTTG
P69	CTGTTGTAGTTGTTGCTGGTAAAG
P173	ACTATGACTATGTTGTCAGTTCACATTCTCATGATCAGAACCTAATTTTACTATAA
P174	TGATCATGAGAATGTGAACTGACAACATAGTCATAGTCAAAAACATTACTTTAAGGAACA
P183	TCTTGAGCTGCTACAGCTGGTTAGATTCTGATTTTTTTTTTTTTTGGAAATCCAATAAATT
P184	TCAGAATCTAACCAGCTGTAGCAGCTCAAGAGAAGCGTTGATTTTTGTAACGATAAA
P93	AACCCCAAGCCCTCAAGATT
P96	GGCAGTTGTCTATAATCGTTCTAA
P38	ATGATAGAAGTTTTAATGATAGAAGATGA
P40	ATCCATAGTTATAAAGCATCTAAAAAAGATAGAGAAACGCAA
P41	CAGTTTGTCGCACTGATAATTCGCTCAAGGGGATAA
P43	ACCTGTTTGTCATCGCTGTATTTGA
P59	ACGTTTGGGAAGTATTATGAGGA
**qPCR analysis**	
sabA-qP1F	TGAACGAAAAATACGAGCAA
sabA-qP1R	CCCGCATAAAGACTCCAAAA
sabA-qP2F	TTCGGTATTGGGTTTTTGGAG
sabA-qP2R	GCTTTAGTGGTAGCGTTTTGT
ppk-2F	GCAAACCCAAAAAGGGAGTCA
ppk-2R	TGACCAATTCCCACAAATCCC
alpB-1F	TTGGGCAATTTGATTGATTTGTATC
alpB-1R	CTCACTGAGCTGGTTGGA
ureA-1F	AAGTTGATGCTCCACTACGC
ureA-1R	CCCTTCTTGCATCAATTCAGC
hup-1F	AATACAACAGCAAAAGAGAAGCC
hup-1R	GCACTCGTTTGTCTTCAGTTTTA
gyrA-1F	GCTAGGATCGTGGGTGATGT
gyrA-1R	TGGCTTCAGTGTAACGCATC
rrnA-2F	CTCCCTACGGGGGAAAGATT
rrnA-2R	GTAGGAGTCTGGACCGTGTC
**Primer extension, EMSA and DNase I Footprint**	
sabA-8	CTTTTTCATAAAAGTGTTCCTT
485	CGAGAGATATTCGTGGCGTTC
486	GCGTTGGCCGATTCATTAATGC
sabA-5	CGTTCTTGTTTTAGTTTGGC

Table S3. Strains used for sequence alignment of region 2-4 of P*sabA* shown in Fig. S2A.

Strain name	Genbank	Strain name	Genbank
35A	CP002096.1	OKI112	CP006821.1
51	CP000012.1	OKI128	CP006822.1
83	CP002605.1	OKI154	CP006823.1
98-10	ABSX00000000.1	OKI422	CP006824.1
B8	FN598874.1	OKI673	CP006825.1
B38	FM991728.1	OKI828	CP006826.1
B128	CP024951.1	OKI898	CP006827.1
BM012A	CP006888.1	PeCan4	CP002074.1
BM013A	CP007604.1	PeCan18	CP003475.1
Cuz20	CP002076.1	Puno120	CP002980.1
ELS37	CP002953.1	Puno135	CP002982.1
F16	AP011940.1	Sat464	CP002071.1
F30	AP011941.1	Shi112	CP003474.1
F32	AP011943.1	Shi169	CP003473.1
F57	AP011945.1	Shi417	CP003472.1
G27	CP001173.1	Shi470	CP001072.1
Gambia94/24	CP002332.1	SJM180	CP002073.1
HPAG1	CP000241.1	SNT49	CP002983.1
India7	CP002331.1	UM032	CP005490.1
J166	CP007603.1	UM037	AUSI00000000.1
Lithuania75	CP002334.1	UM066	AUSJ00000000.1
OK113	AP012600.1	UM298	CP006610.2
OK310	AP012601.1	UM299	CP005491.3
OKI102	CP006820.1	V225d	CP001582.1

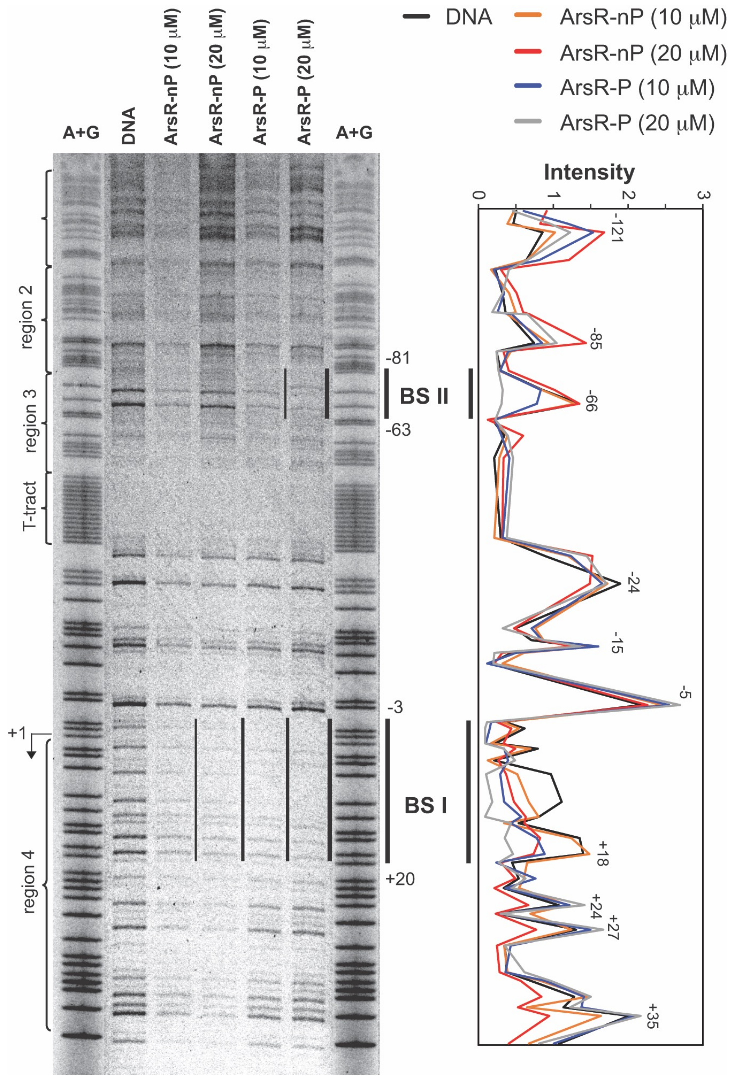

A+G
DNA
ArsR-nP (10 μM)
ArsR-nP (20 μM)
ArsR-P (10 μM)
ArsR-P (20 μM)
A+G
region 2
region 3
T-tract
region 4
+1
-81
-63
-3
+20
BS II
BS I
DNA
ArsR-nP (10 μM)
ArsR-nP (20 μM)
ArsR-P (10 μM)
ArsR-P (20 μM)
Intensity
0
1
2
3
-121
-85
-66
-24
-15
-5
+18
+24
+27
+35

Fig. S1

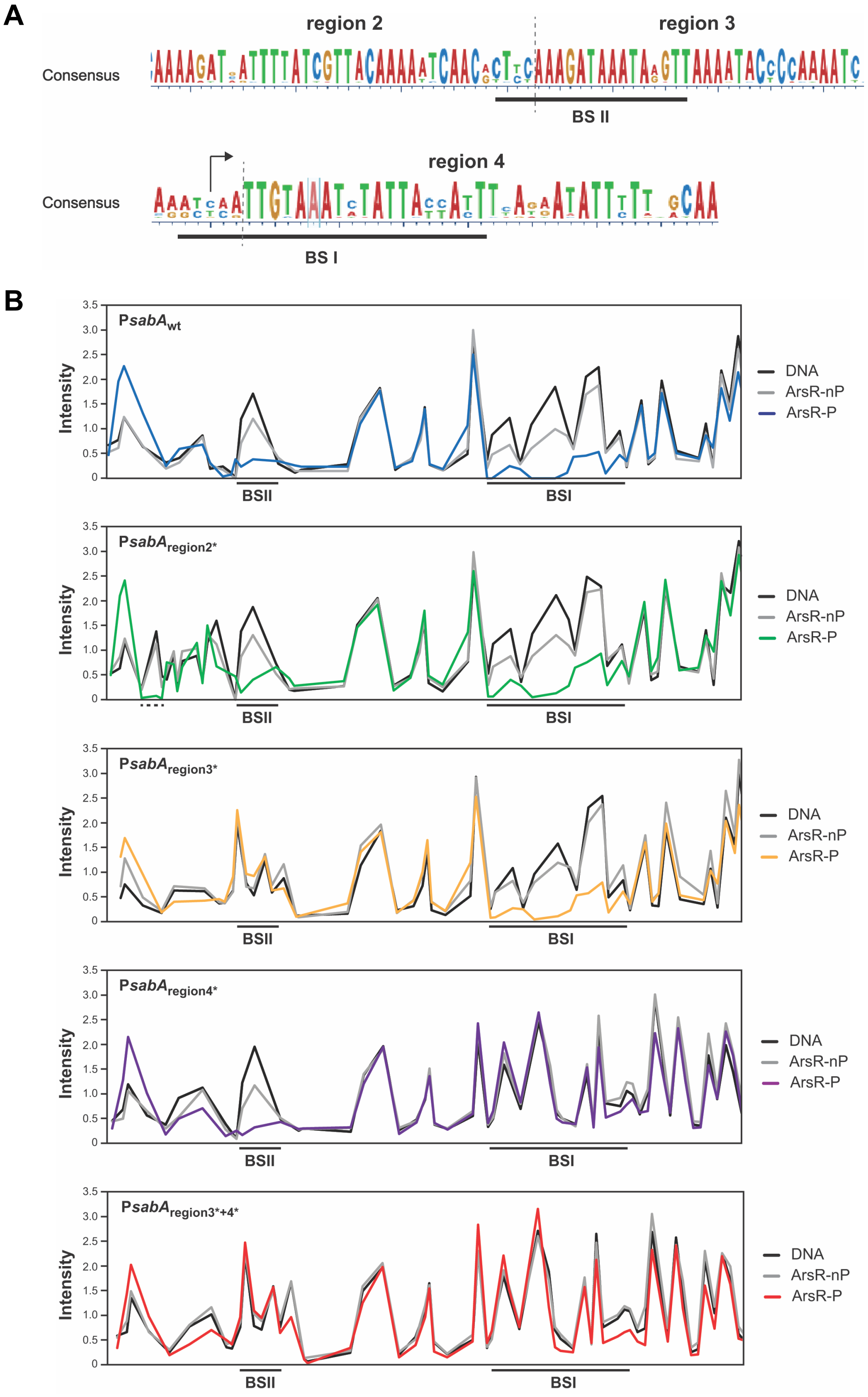
A
region 2
region 3
Consensus
BS II
region 4
Consensus
BS I
B
PsabAwt
Intensity
DNA
ArsR-nP
ArsR-P
BSII
BSI
PsabAregion2*
PsabAregion3*
PsabAregion4*
PsabAregion3*+4*

Fig. S2

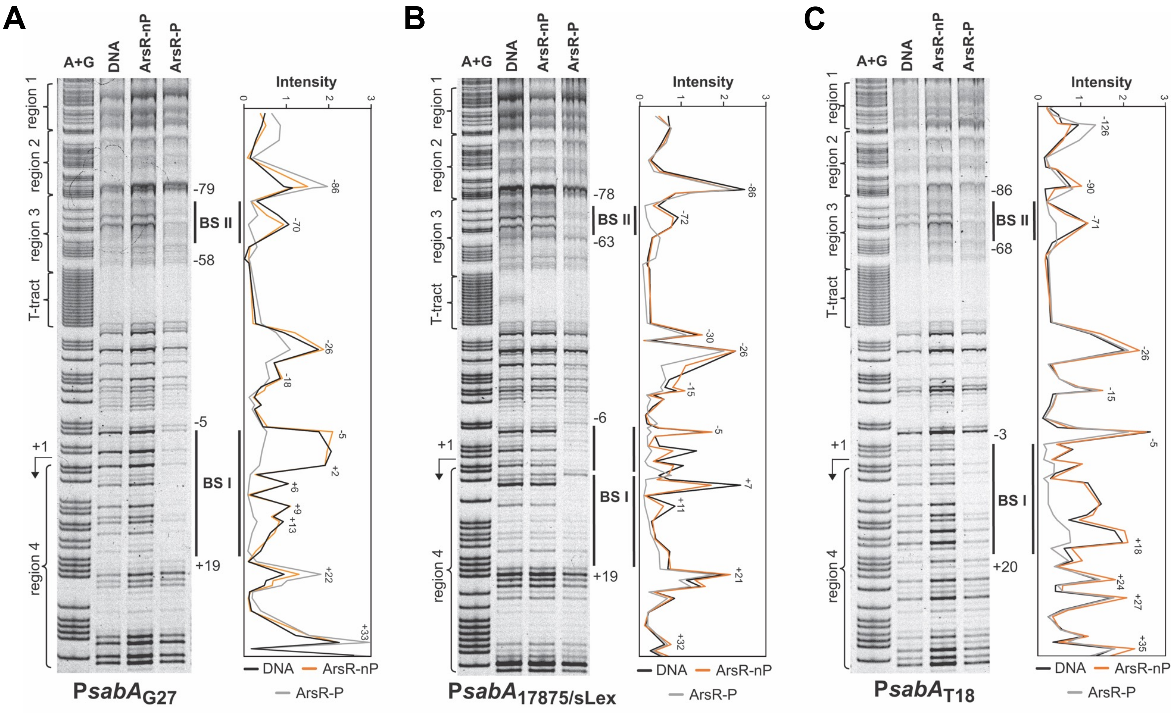
A
A+G
DNA
ArsR-nP
ArsR-P
region 1
region 2
region 3
T-tract
region 4
+1
-79
BS II
-58
-5
BS I
+19
Intensity
DNA
ArsR-nP
ArsR-P
PsabA G27
B
-78
-63
-6
+19
PsabA 17875/sLex
C
-86
-68
-3
+20
PsabA T18

Fig. S3

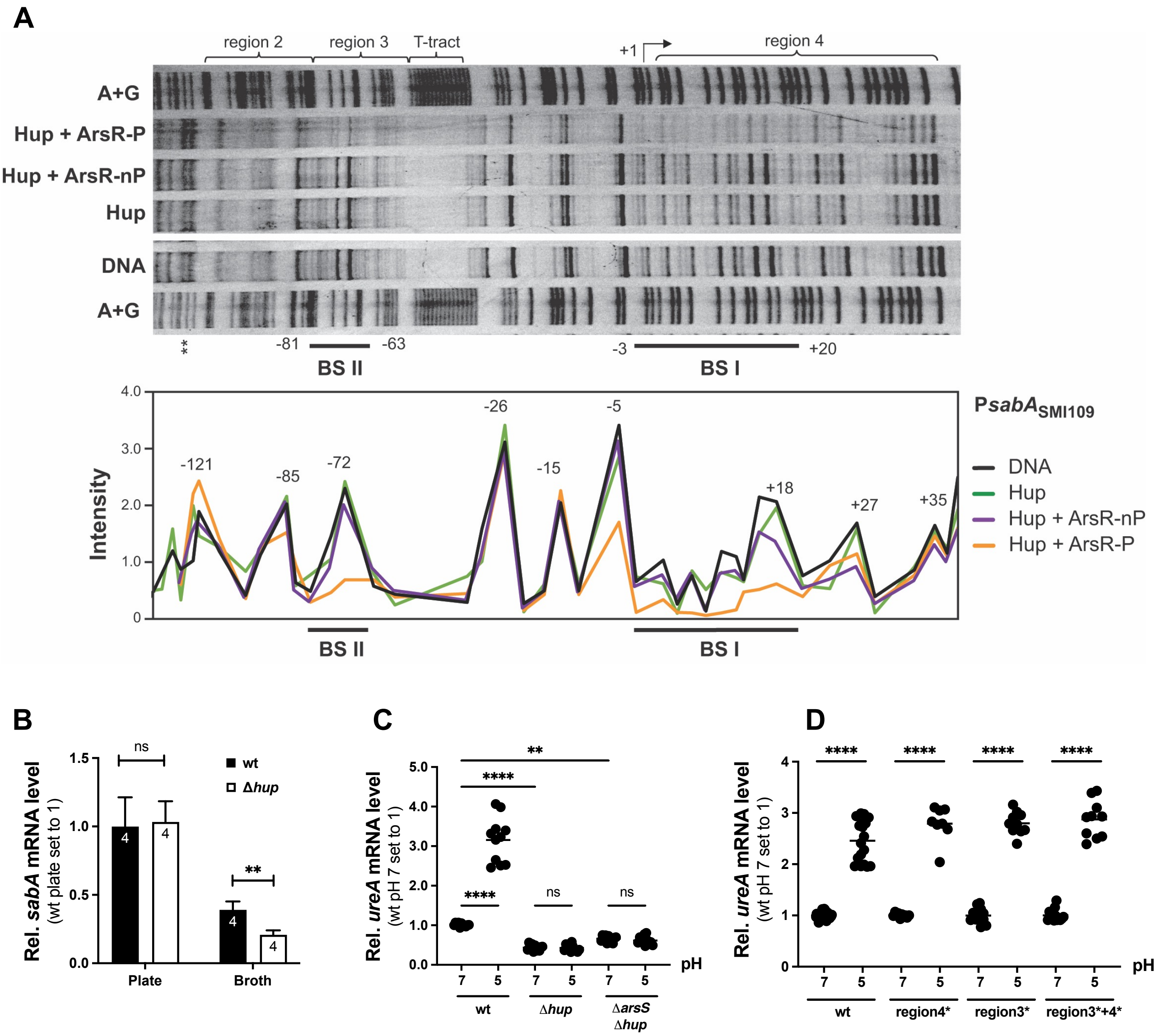
A
region 2
region 3
T-tract
+1
region 4
A+G
Hup + ArsR-P
Hup + ArsR-nP
Hup
DNA
A+G
-81
-63
BS II
-3
+20
BS I
PsabA SMI109
Intensity
DNA
Hup
Hup + ArsR-nP
Hup + ArsR-P
-121
-85
-72
-26
-15
-5
+18
+27
+35
BS II
BS I
B
Rel. sabA mRNA level
(wt plate set to 1)
wt
Δhup
ns
**
Plate
Broth
C
Rel. ureA mRNA level
(wt pH 7 set to 1)
**
****
****
ns
ns
pH
wt
Δhup
ΔarsS Δhup
D
Rel. ureA mRNA level
(wt pH 7 set to 1)
****
pH
wt
region4*
region3*
region3*+4*

Fig. S4

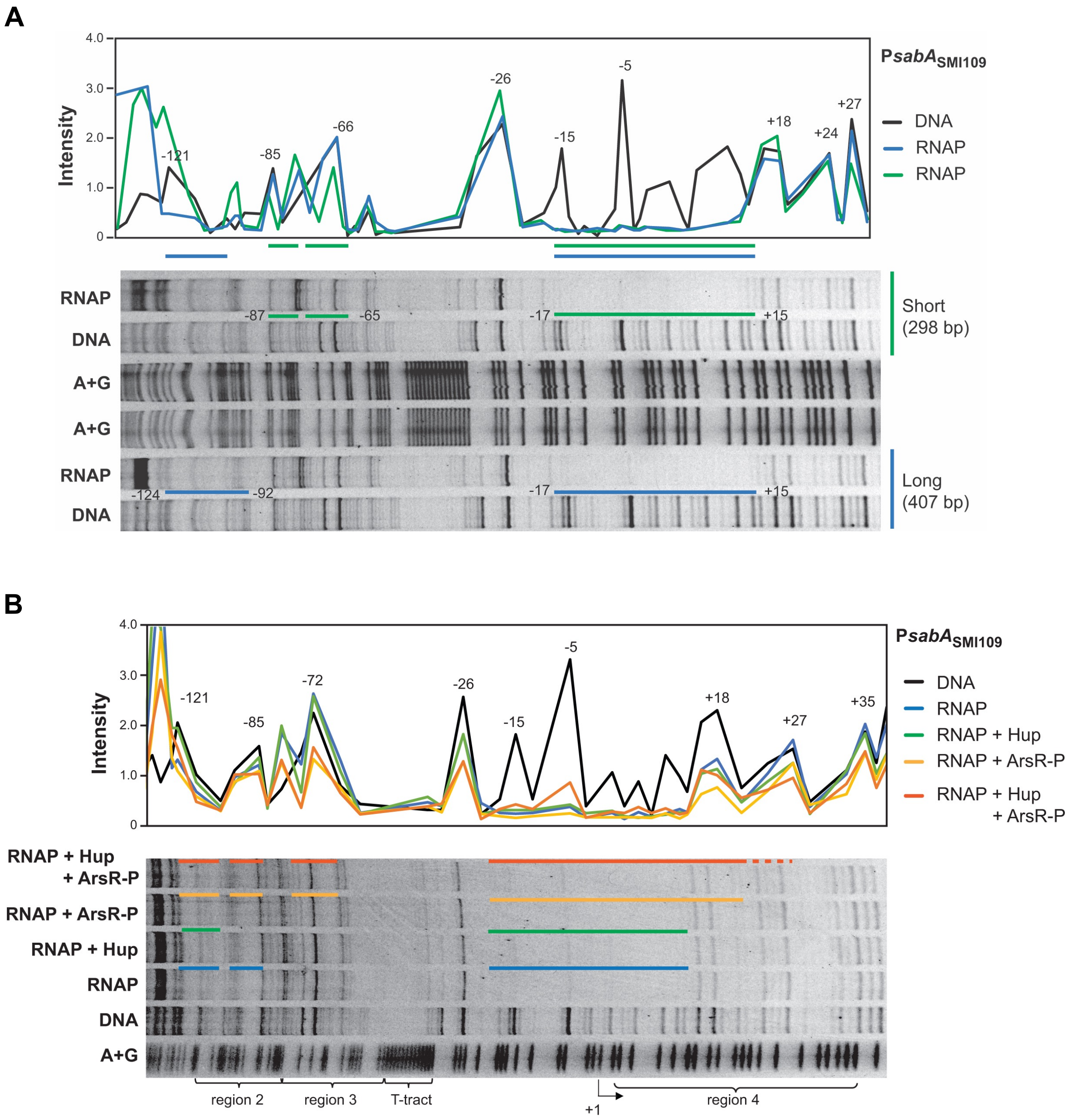

A
PsabA SMI109
Intensity
DNA
RNAP
RNAP
-121
-85
-66
-26
-15
-5
+18
+24
+27
RNAP
DNA
A+G
A+G
RNAP
DNA
-87
-65
-17
+15
-124
-92
Short (298 bp)
Long (407 bp)
B
PsabA SMI109
Intensity
DNA
RNAP
RNAP + Hup
RNAP + ArsR-P
RNAP + Hup + ArsR-P
-121
-85
-72
-26
-15
-5
+18
+27
+35
RNAP + Hup + ArsR-P
RNAP + ArsR-P
RNAP + Hup
RNAP
DNA
A+G
region 2
region 3
T-tract
+1
region 4

Fig. S5

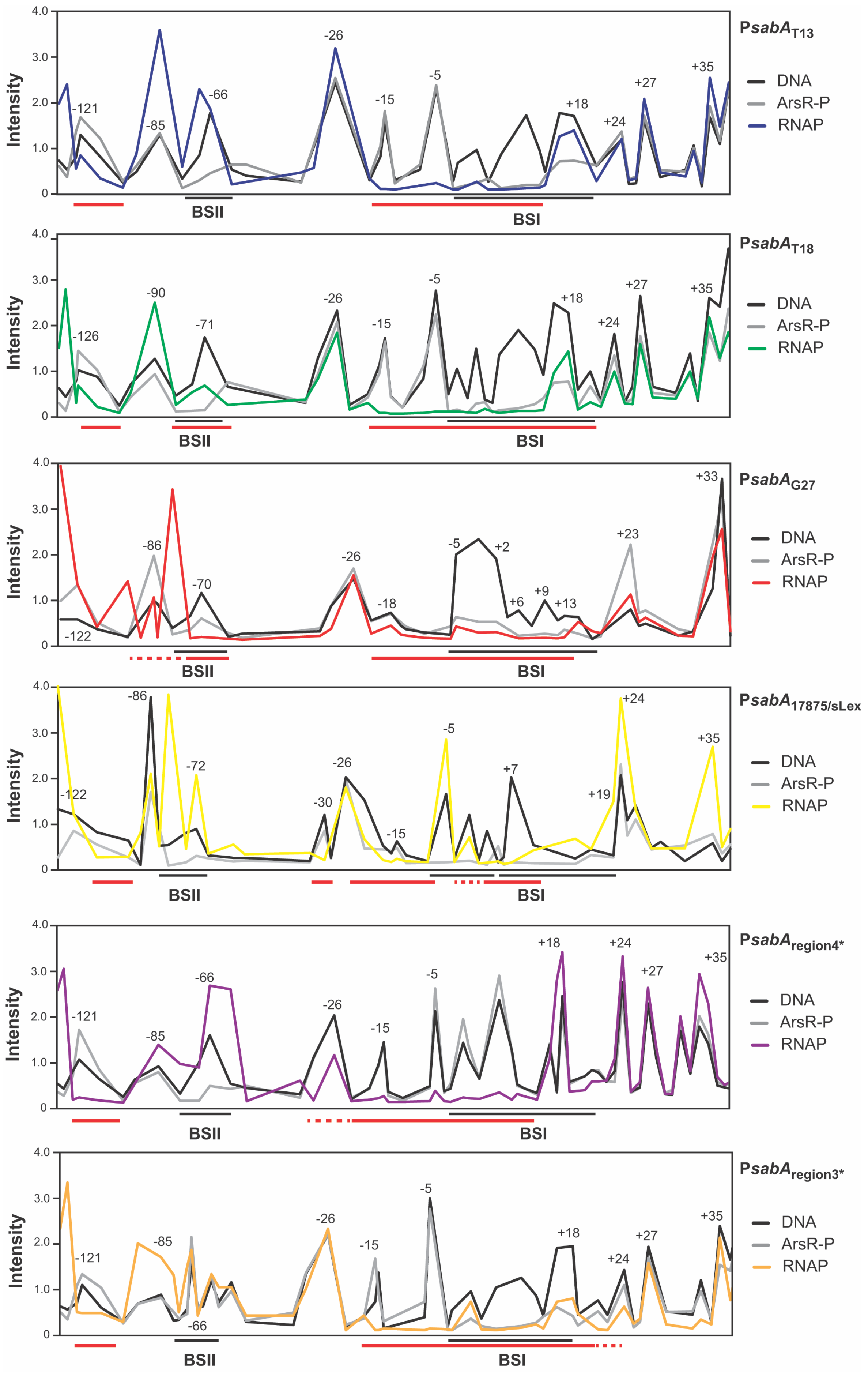
PsabA$_{T13}$
PsabA$_{T18}$
PsabA$_{G27}$
PsabA$_{17875/sLex}$
PsabA$_{region4*}$
PsabA$_{region3*}$
DNA
ArsR-P
RNAP
Intensity
BSII
BSI

Fig. S6

Figure S1. ArsR binding to P*sabA* DNA occurs at two different sites.

Binding of His_6-ArsR to P*sabA* DNA analyzed by EMSA followed by DNase I footprint analysis. A total of 25 nM P*sabA* DNA (407 bp, the same as in Fig. 2A) was mixed with protein storage buffer (lane DNA), 10–20 µM ArsR-nP (lane ArsR-nP), or 10–20 µM ArsR-P (lane ArsR-P) for 30 min at 25°C. After DNase I treatment, samples were run in TBE PAGE, and non-shifted and shifted DNA bands were cut from the gel and purified and then loaded onto a sequencing gel. Transcriptional start site (+1) and T-tract are indicated along the left side, as well as the regions described in Fig. 3A. Binding sites are marked by solid lines, and the nucleotide positions of the binding sites are shown along the right side. The image shown is one representative of two independent experiments. Line representations of the protection pattern of ArsR-nP and ArsR-P on P*sabA* are shown in the diagram to the right. The densities of the DNase footprint bands (indicated by intensity) are plotted as a migration (from the top to bottom of the DNase footprint). Numbers representing nucleotide positions relative to the transcriptional start site (+1) are shown on top of the peaks. The ArsR BS I and BS II found by DNase I footprinting (Fig. 2C-D) are shown as solid black lines below the diagram.

Figure S2. Binding of ArsR to the two sites in P*sabA* DNA is affected by AT-rich DNA sequences.

(A) Sequence alignment of region 2-4 of the *sabA* promoter in 48 different *H. pylori* strains with annotated sequences (see Table S3). The regions described in Fig. 3A are separated by vertical lines and noted above the alignement, and the solid black lines mark the ArsR BS I and BS II found by DNase I footprinting. **(B)** Line representation of the protection pattern of ArsR-nP and ArsR-P on P*sabA* with scrambled region 2*, 3*, 4*, and 3*+4*. The densities of the DNase footprint bands (indicated by intensity) are plotted as a migration (from the top to bottom of the DNase footprint). Each diagram represents different P*sabA* DNA templates with the DNase I footprint result for DNA alone, ArsR-nP, and ArsR-P as shown in Fig. 3B-C. The positions of ArsR BS I and BS II found by DNase footprinting are shown as solid black lines below the diagrams.

Figure S3. Binding of ArsR to P*sabA* DNA from different strains or different T-tract lengths.

DNase I footprint analysis of ArsR binding to P*sabA* DNA (410 bp PCR; primers 486/485-TET) from **(A)** G27 (template pAAG265), **(B)** 17875/sLex (template pAAG266), and **(C)** the isogenic T_{18} variant of SMI109 (template pAAG286). Transcriptional start site (+1), T-tract, and the regions described in Fig. 3A are shown to the left of the gel images. Maxam and Gilbert DNA sequencing reaction (lane A+G) shows the sequences of the DNA used. DNA was mixed with buffer (lane DNA), 10 µM ArsR-nP (lane ArsR-nP), or 10 µM ArsR-P (lane ArsR-P). Binding sites are marked by solid lines, and nucleotide positions are shown to the right of each gel image. The images show one representative gel of at least two independent experiments. A line representation of the protection pattern of ArsR is shown in the diagram to the right of each gel image (black, free DNA; orange, ArsR-nP; grey, ArsR-P). The densities of the DNase I footprint bands (indicated by intensity) are plotted as a migration (from the top to bottom of the DNase footprint). Nucleotide positions are written on top of the peaks, and the two ArsR binding sites are marked by solid lines.

Figure S4. Expression of control genes in SMI109 wt and in strains with scrambled regions of the *sabA* promoter or in SMI109 Δ*hup* strains.

(A) DNase I footprint analysis or Hup-His_6 and/or His_6-ArsR binding to P*sabA* DNA from SMI109. A total of 25 nM DNA (407 bp PCR; primers 486/485-TET; template pAAG264) was mixed with protein storage buffer (lane DNA), 5 µM Hup-His (lane Hup), and 10 µM ArsR-nP (lane Hup + ArsR-nP) or 10 µM ArsR-P (lane Hup + ArsR-P). Transcriptional start site (+1) and T-tract are marked on top of the gel image, as well as the regions described in Fig. 3A. The Maxam and Gilbert DNA sequencing reaction (lane A+G) shows the sequence of the DNA used. Binding sites are marked by solid lines, and the nucleotide positions of the binding sites are shown below the gel image. The image shows one representative gel of at least two independent experiments. Line representations of the protection pattern of Hup alone, Hup and ArsR-nP or Hup and ArsR-P are shown below the image. The densities of the DNase footprint bands (indicated by intensity) are plotted as a migration (from the top to bottom of the DNase footprint). Nucleotide positions are written on top of the peaks in each diagram. The ArsR binding sites I and II are shown as solid black lines. **(B)** Expression of *sabA* analyzed by RT-qPCR after growth on a plate or in broth in SMI109 wt and Δ*hup* strains. Samples were collected after overnight growth on blood agar plates or from bacteria grown in Brucella broth to an OD_{600} of 0.3. Data from two biological replicates and two technical replicates for each biological replicate were used for the quantification. The number of data points for each average are written on the bars of the diagram. Statistical analysis was performed with Mann–Whitney U-tests ($p < 0.01$, **; $p > 0.05$, ns). **(C-D)** The effect of acid stress on *ureA* mRNA levels as analyzed by RT-qPCR. SMI109 wt, Δ*hup*, and Δ*hup* Δ*arsS* (C) or isogenic variants of SMI109 with scrambled P*sabA* DNA regions 3-4 (D). Cultures were grown in Brucella broth to an OD_{600} of 0.3 before the shift to pH 5 or grown at pH 7 as a control. Samples were collected 24 h after the pH shift for mRNA level analysis. Data from at least two biological replicates, each including two technical replicates, were used for the quantifications. The number of samples included are shown as separate dots in the diagram. Statistical analysis was performed using Mann–Whitney U-tests ($p < 0.0001$, ****; $p < 0.01$, **; $p > 0.05$, ns).

Figure S5. Binding of RNAP to P*sabA* DNA of different lengths and together with Hup-His_6 and/or His_6-ArsR.

(A) Binding of RNAP to P*sabA* DNA of different lengths was analyzed by DNase I footprinting. A total of 25 nM TET-labelled P*sabA* DNA was mixed with protein storage buffer (lane DNA) or 300 nM *E. coli* σ^{70}-RNAP (lane RNAP). PCR-generated DNA was used at two different lengths: long (407 bp, 486/485-TET primers, blue) or short (298 bp, SabA-5/485-TET primers, green). Maxam and Gilbert DNA sequencing reaction (lane A+G) shows the sequences of the DNA used. A line representation of the protection pattern of RNAP on P*sabA* is shown in the diagram; DNA (black), RNAP + long DNA (blue), and RNAP + short DNA (green). The densities of the DNase footprint bands (indicated by intensity) are plotted as a migration (from the top to bottom of the DNase footprint). Nucleotide positions are written on top of the peaks in the diagram. The RNAP αCTD and σ^{70}-subunit binding sites are shown as solid lines, and nucleotide positions are written on the gel. **(B)** Binding of RNAP together with Hup-His_6 and/or His_6-ArsR to P*sabA* DNA from SMI109 was analyzed by DNase I footprinting. A total of 25 nM DNA

(407 bp PCR; primers 486/485-TET; template pAAG264) was mixed with protein storage buffer (lane DNA), 150 nM *E. coli* σ^{70}-RNAP (lane RNAP), RNAP + 5 µM Hup-His (lane RNAP+Hup), RNAP + 10 µM ArsR-P (lane RNAP+ArsR-P) or both (RNAP+Hup+ArsR-P). Transcriptional start site (+1) and T-tract are marked at bottom of the gel image, as well as the regions described in Fig. 3A. The Maxam and Gilbert DNA sequencing reaction (lane A+G) shows the sequence of the DNA used. The image shows one representative gel of at least two independent experiments. Line representations of the protection pattern is shown in the diagram above the gel image. The densities of the DNase footprint bands (indicated by intensity) are plotted as a migration (from the top to bottom of the DNase footprint). Nucleotide positions are written on top of the peaks in each diagram. The detected protected regions in each sample are shown as colored lines on the gel image.

Figure S6. Binding of RNAP to P*sabA* DNA from different strains with different T-tract lengths and scrambled region 3* and 4*.
Line representations of the protection pattern of ArsR-P or RNAP on P*sabA* from the SMI109 T_{13} (wt) and SMI109 T_{18} variant, G27, and 17875/sLex or region 4* and region 3*. The densities of the DNase footprint bands (indicated by intensity) are plotted as a migration (from the top to bottom of the DNase footprint). Each diagram represents different P*sabA* DNA templates with the DNase I footprint result for DNA alone, ArsR-P, or RNAP as shown in Fig. 8A-C. The sequences of the DNAs that were used are shown in Fig. 3A. Nucleotide positions are written on top of the peaks in each diagram. The ArsR binding sites I and II are shown as solid black lines, and the binding sites of RNAP are indicated by red lines below each diagram.

References for Supplement

1. Baltrus, D.A., Amieva, M.R., Covacci, A., Lowe, T.M., Merrell, D.S., Ottemann, K.M., Stein, M., Salama, N.R. and Guillemin, K. (2009) The complete genome sequence of *Helicobacter pylori* strain G27. *J Bacteriol*, **191**, 447-448.
2. Olofsson, A., Vallström, A., Petzold, K., Tegtmeyer, N., Schleucher, J., Carlsson, S., Haas, R., Backert, S., Wai, S.N., Grobner, G. *et al.* (2010) Biochemical and functional characterization of *Helicobacter pylori* vesicles. *Mol Microbiol*, **77**, 1539-1555.
3. Mahdavi, J., Sondén, B., Hurtig, M., Olfat, F.O., Forsberg, L., Roche, N., Ångström, J., Larsson, T., Teneberg, S., Karlsson, K.A. *et al.* (2002) *Helicobacter pylori* SabA adhesin in persistent infection and chronic inflammation. *Science*, **297**, 573-578.
4. Åberg, A., Gideonsson, P., Vallström, A., Olofsson, A., Öhman, C., Rakhimova, L., Borén, T., Engstrand, L., Brannström, K. and Arnqvist, A. (2014) A repetitive DNA element regulates expression of the *Helicobacter pylori* sialic acid binding adhesin by a rheostat-like mechanism. *PLoS Pathog*, **10**, e1004234.
5. Yanisch-Perron, C., Vieira, J. and Messing, J. (1985) Improved M13 phage cloning vectors and host strains: nucleotide sequences of the M13mp18 and pUC19 vectors. *Gene*, **33**, 103-119.
6. Datsenko, K.A. and Wanner, B.L. (2000) One-step inactivation of chromosomal genes in *Escherichia coli* K-12 using PCR products. *Proc Natl Acad Sci U S A*, **97**, 6640-6645.
7. de Vries, N., Kuipers, E.J., Kramer, N.E., van Vliet, A.H., Bijlsma, J.J., Kist, M., Bereswill, S., Vandenbroucke-Grauls, C.M. and Kusters, J.G. (2001) Identification of environmental stress-regulated genes in *Helicobacter pylori* by a *lacZ* reporter gene fusion system. *Helicobacter*, **6**, 300-309.
8. Elliott, T. and Geiduschek, E.P. (1984) Defining a bacteriophage T4 late promoter: absence of a "-35" region. *Cell*, **36**, 211-219.
